# Synthesis and structure-activity relationships for tetrahydroisoquinoline-based inhibitors of *Mycobacterium tuberculosis*

**DOI:** 10.1016/j.bmc.2020.115784

**Published:** 2020-11-15

**Authors:** Guo-Liang Lu, Amy S.T. Tong, Daniel Conole, Hamish S. Sutherland, Peter J. Choi, Scott G. Franzblau, Anna M. Upton, Manisha U. Lotlikar, Christopher B. Cooper, William A. Denny, Brian D. Palmer

**Affiliations:** a Auckland Cancer Society Research Centre, School of Medical Sciences, Private Bag 92019, Auckland 1142, New Zealand; b Maurice Wilkins Centre, University of Auckland, Private Bag 92019, Auckland 1142, New Zealand; c Institute for Tuberculosis Research, College of Pharmacy, University of Illinois at Chicago, 833 South Wood Street, Chicago, IL 60612, USA; d Global Alliance for TB Drug Development, 40 Wall St, NY 10005, USA

**Keywords:** ATP synthase, Structure-activity relationships, Synthesis, Tetrahydroquinolines, Tuberculosis, DCM, dichloromethane, DIPEA, *N*,*N*-diisopropylethylamine, Et_2_O, diethyl ether, EtOAc, ethyl acetate, THF, tetrahydrofuran, MeOH, methanol

## Abstract

A series of 5,8-disubstituted tetrahydroisoquinolines were shown to be effective inhibitors of *M. tb* in culture and modest inhibitors of *M. tb* ATP synthase. There was a broad general trend of improved potency with higher lipophilicity. Large substituents (e.g., Bn) at the tetrahydroquinoline 5-position were well-tolerated, while *N*-methylpiperazine was the preferred 8-substituent. Structure-activity relationships for 7-linked side chains showed that the nature of the 7-linking group was important; –CO– and –COCH_2_– linkers were less effective than –CH_2_– or –CONH– ones. This suggests that the positioning of a terminal aromatic ring is important for target binding. Selected compounds showed much faster rates of microsomal clearance than did the clinical ATP synthase inhibitor bedaquiline, and modest inhibition of mycobacterial ATP synthase.

## Graphical abstract

**Figure:**
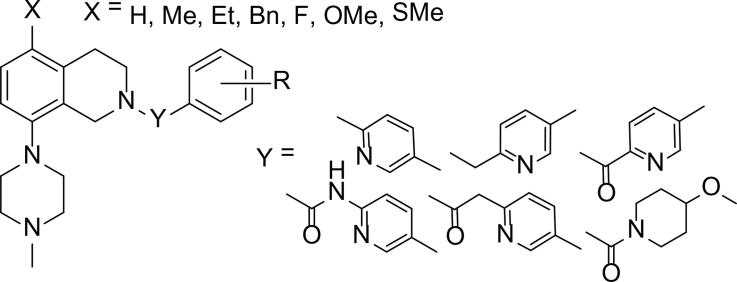

## 1 Introduction

**Fig. 1:**
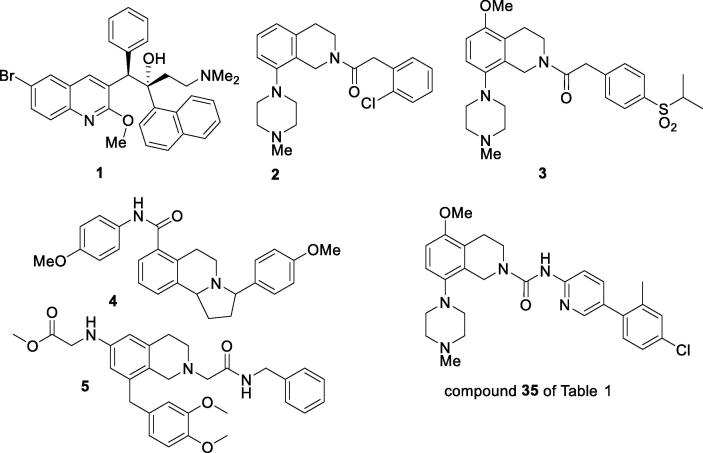
Examples of bioactive *N*-substituted tetrahydroisoquinolines.

The treatment of drug-resistant tuberculosis (TB) has in recent times become a major global health problem.^1^ Recent encouragement has come from the discovery^2^ of the drug bedaquiline (**1**) (Fig. 1), which is a selective inhibitor of the ATP synthase enzyme of the causal bacterium *Mycobacterium tuberculosis* (*M.tb*), and is clinically effective against drug-resistant strains.^3^ A “second-generation” analogue of bedaquiline (TBAJ-876) has recently began clinical trial.^4^, ^5^, ^6^, ^7^

In a search for new drugs for the treatment of tuberculosis we report here, from a screening lead (compound **13** of Table 1), the synthesis and structure–activity relationships of a new class of *N*-substituted 5,8-disubstituted tetrahydroisoquinolines and develop initial structure–activity relationships for a series of amide and urea analogues of these. *N*-Substituted tetrahydroisoquinolines have previously been reported as antagonists of the 5-hydroxytryptamine 5HT_1B_ receptor^8^ (e.g., **2,** Fig. 1), as potential anti-depressive agents^9^ (e.g., **3**) and as anti-hypertensive agents^10^ (e.g., **4**), and as inhibitors of the excitatory neuropeptides orexin-A and orexin-B (e.g., **5**),^11^ but have not been reported as anti-tubercular agents.

**Table 1:** Structural and biological data for 5, *N*-disubstituted 8-(4-methylpiperazin-1-yl)-1,2,3,4-tetrahydroisoquinolines

				MIC		IC_50_	
No	X	Y	R	MABA^a^	LORA^b^	VERO^c^	clogP^d^
**6**	Me		2-Me, 4-Cl	1.9	2.4	22	6.03
**7**	Me		4-tBu	1.4	3.4	18	7.12
**8**	OMe		2-Me, 4-Cl	1.9	3.6	12	5.43
**9**	OMe		3,5-diCF_3_	7.9	12	19	6.31
**10**	H		4-Cl	1.8	16	16	5.17
**11**	H		2-Me, 4-Cl	3.6	11	15	5.36
**12**	Me		2-Me, 4-Cl	0.79	5.2	14	5.86
**13**	OMe		2-Me, 4-Cl	1.8	6.1	12	5.27
**14**	SMe		2-Me, 4-Cl	3.4	6.0	11	5.81
**15**	F		2-Me, 4-Cl	3.8	6.1	24	5.68
**16**	F		2,4-diF	15	21	22	5.06
**17**	F		2-CF_3_, 4-Cl	2.3	5.4	11	5.65
**18**	H		2-Me, 4-Cl	11	13	20	4.80
**19**	H		4-Cl	16	20	27	4.60
**20**	H		3-Cl	15	11.3	24	4.60
**21**	OMe		4-Cl	13	12	11	4.51
**22**	OMe		2-Me, 4-Cl	8.9	8.0	22	5.30
**23**	H		4-OCF_3_	3.6	3.9	14	5.02
**24**	H		2-Me, 4-Cl	6.2	7.7	11	4.83
**25**	Me		2,4-diCl	1.95	1.99	12	5.60
**26**	Me		3,5-diCF_3_	3.1	5.3	>32	6.22
**27**	Et		4-CF_3_	1.0	0.9	12	5.73
**28**	Et		2-Me, 4-Cl	2.9	1.8	12	5.74
**29**	Bn		2-Me, 4-Cl	1.8	1.5	11	7.08
**30**	Bn		2,4-diMe	2.0	1.9	11	6.66
**31**	OMe		3,5-diaza	>32	>32	>32	2.59
**32**	OMe		3-aza, 4-OMe	15.5	20	14	3.30
**33**	OMe		4-OMe	7.1	7.4	16	3.82
**34**	OMe		2-Cl, 4-CF_3_	3.6	3.2	12	5.19
**35**	OMe		2-Me, 4-Cl	1.9	3.1	12	4.73
**36**	OMe		2,4-diCl	1.9	ND^e^	>32	5.01
**37**	OMe		4-OCF_3_	3.1	3.0	12	4.93
**38**	OMe		3,5-diCF_3_	1.3	ND^e^	10	6.67
**39**	SMe		3,5-diCF_3_	3.6	4.0	20	6.17
**40**	SMe		2-Me, 4-Cl	1.9	2.0	13	5.28
**41**	F		3-CF_3_, 4-Cl	1.4	2.9	10	6.18
**42**	F		2-Me, 4-Cl	1.2	5.4	12	5.14
**43**	H		4-Cl	14.1	20.3	19	4.05
**44**	H		H	>32	>32	>32	3.32
**45**	H		3-Cl	15.0	21.7	25	4.05
**46**	H		4-OCF_3_	13.7	12.4	20	4.45
**47**	H		2-Me, 4-Cl	14.0	19.4	11	4.25
**48**	Me		4-OCF_3_	6.4	3.9	19	5.71
**49**	Me		2-CF_3_	7.7	7.3	11	5.64
**50**	OMe		4-OCF_3_	7.6	7.0	15	5.12
**51**	OMe		2-CF_3_	12	10.0	24	5.05

^b^MIC_90_ (µg/mL); minimum inhibitory concentration for inhibition of growth of *M.tb* strain H37Rv, determined under aerobic (replicating; MABA)^12^ or non-replicating (LORA)^13^ conditions, determined at the Institute for Tuberculosis Research, University of Illinois at Chicago. ^c^Cytotoxicity (IC_50_, µg/mL) in Vero green monkey-derived epithelial kidney cells;^14^^d^clogP calculated by ChemDraw Ultra v12.0.2. (CambridgeSoft); ^e^Not done.

## 2 Results and discussion

### 2.1 Chemistry

**Scheme 1:**
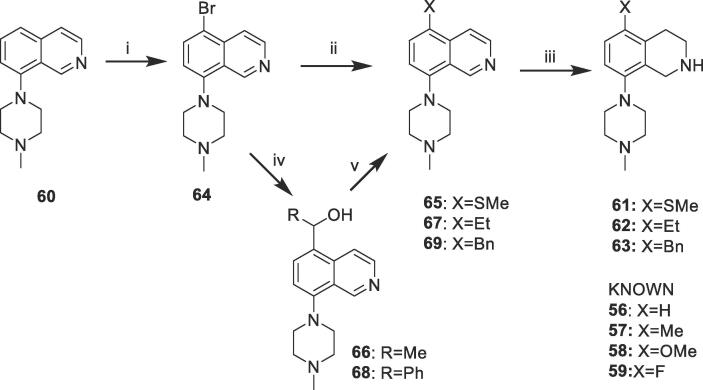
Syntheses of 5-substituted tetrahydroisoquinoline intermediates.

The tetrahydroisoquinolines of Table 1 were prepared from the appropriate 5-substituted-8-bromoisoquinolines. Seven different 5-substituents were evaluated, and four of these (X = H, Me, OMe, F; compounds **56**–**59** respectively) were commercially available. The SMe-, Et- and Bn-substituted analogues (**61**–**63**) were prepared from the known isoquinoline **60** as shown in Scheme 1. Bromination of **60** with NBS gave the 5-Br compound **64**, which was thiomethylated to give **65**, then reduced to give **61**. Lithiation of **64** and treatment with acetaldehyde gave **66**, which was successively reduced with Et_3_SiH to give **67**, then with NaCNBH_3_ to **62**. Similar condensation of **64** with benzaldehyde gave **68**, which was similarly reduced via **69** to give **63**.

*Reagents and conditions*: (i) NBS, DMF, 20 °C, 3 days; (ii) nBuLi, THF, −78 °C, 5 min, then MeSSMe, −78 °C, 2 h; (iii) NaCNBH_3_, BF_3_.Et_2_O, MeOH, reflux, 22 h; (iv) nBuLi, THF, −78 °C, 5 min, then RCHO, −78 °C, 2 h; (v) Et_3_SiH, TFA, 75 °C, 1 h.

**Scheme 2:**
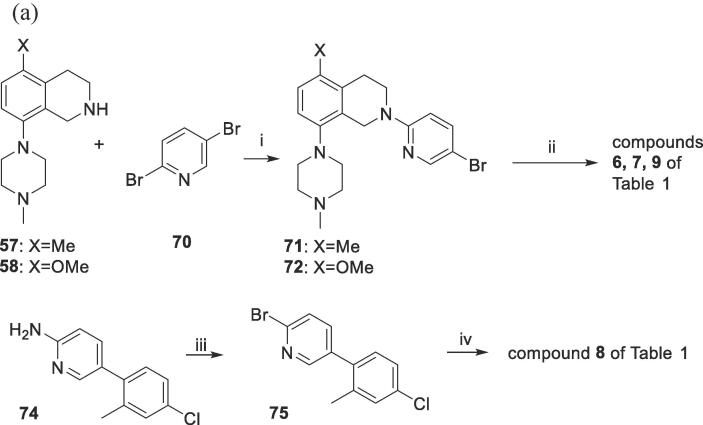

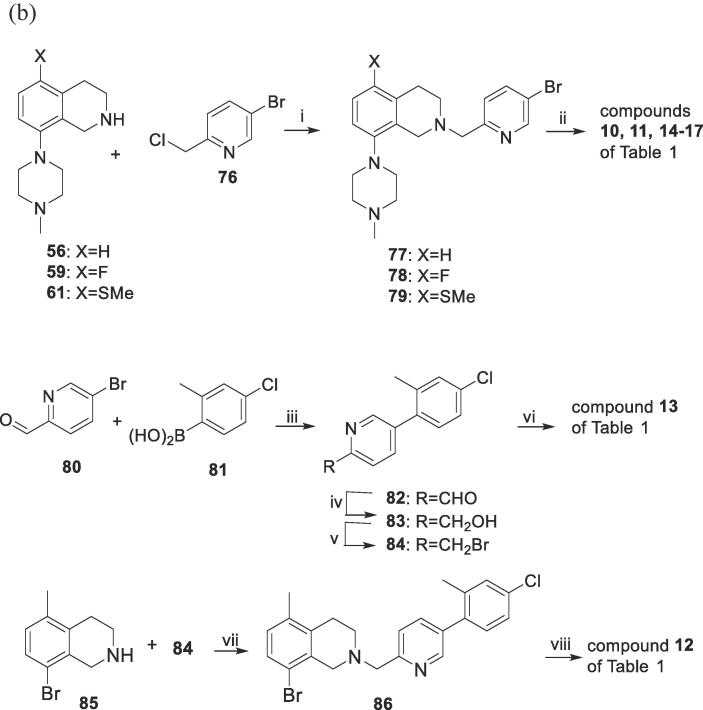
Syntheses of the tetrahydroisoquinolines 6–17 of Table 1.

The compounds of Table 1 were then prepared by elaboration of the various side chains on these tetrahydroisoquinolines. Compounds **6, 7** and **9** (directly linked pyridyl analogues) were synthesized by selective reaction of tetrahydroisoquinolines **57** and **58** with 2,5-dibromopyridine (**70**) to give compounds (**71**, **72**) that were Suzuki-coupled with the appropriate phenylboronic acids (**73**) or **81** (Scheme 2A). Compound **8** was synthesized by Buchwald coupling of **58** with the pre-formed sidechain bromide **75**.

Compounds **10, 11, 14**–**17** (CH_2_-linked analogues) were prepared by reaction of known tetrahydroisoquinolines **56**, **59** and **61** with 5-bromo-2-(chloromethyl)pyridine (**76**) to give intermediates **77**–**79** (Scheme 2B). These were coupled with appropriate phenylboronic acids (**73**) or **81** to give **10**, **11**, **14**–**17** of Table 1. Compound **13** was prepared by the *N*-alkylation reaction of tetrahydroisoquinoline **58** with the fully pre-formed sidechain bromide **84**. This was synthesised by Suzuki coupling of aldehyde **80** and boronic acid **81** to give aldehyde **82**, which was reduced to alcohol **83** and brominated to give **84**. Compound **12** was synthesised by Buchwald coupling of the sidechain-preinstalled tetrahydroisoquinoline-8-bromide **86** with *N*-methylpiperazine. **86** was prepared from tetrahydroisquinoline-8-bromide **85** by the *N*-alkylation with **84**.

2A: directly-linked pyridyl side chains; compounds 6–9 of Table 1

*Reagents and conditions*: (i) Xantphos, Pd_2_(dba)_3_, NaO*^t^*Bu, toluene, 100 °C, 4 h ; (ii) R-PhB(OH)_2_ (**73**) or **81**, 2 M aq Na_2_CO_3_, PdCl_2_dppf, toluene, EtOH, 85 °C, 20 ~ 24 h; (iii) HBr, Br_2_, HCl, NaNO_2_, −5°C; (iv) **58**, BINAP, Pd_2_(dba)_3_, NaO*^t^*Bu, toluene, reflux, 1 h.

2B: –CH*_2_*-linked pyridyl side chains; compounds 10–17 of Table 1

*Reagents and conditions*: (i) K_2_CO_3_, DMF, 20 °C,15 h; (ii) R-PhB(OH)_2_ (**73**) or **81**, aq Na_2_CO_3_, PdCl_2_dppf, toluene, EtOH, 80 °C, various hours; (iii) K_3_PO_4_·H_2_O, water, acetonitrile, dioxane, Pd(PPh_3_)_4_, reflux, 5 h; (iv) NaBH_4_, anhydrous MeOH, 4 h, 20 °C; (v) MsCl, Et_3_N, DCM, 20 °C, 15 min; then LiBr, anhydrous acetone, reflux, 1.5 h; (vi) **58**, K_2_CO_3_, DMF, 20 °C, 5 h (vii) K_2_CO_3_, DMF, 20 °C, 5 h; (viii) *N*-methylpiperazine, BINAP, Pd_2_(dba)_3_, NaO*^t^*Bu, toluene, reflux, 4 h.

**Scheme 3:**
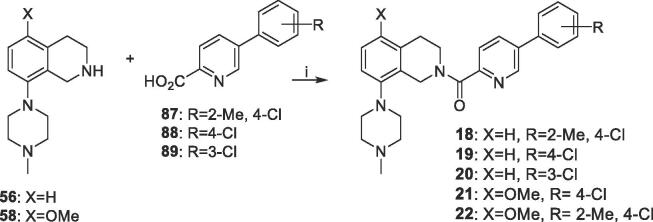
Synthesis of CO-linked compounds 18–22 of Table 1.

The CO-linked compounds **18**–**22** of Table 1 were prepared as shown in Scheme 3, by HATU-mediated coupling of tetrahydroisoquinolines **56** or **58** with commercially-available phenyl-substituted 5-phenylpicolinic acids **(87**–**89**).

*Reagents and conditions*: (i) HATU, DIPEA, DMF, 20 °C, 16 h

**Scheme 4:**
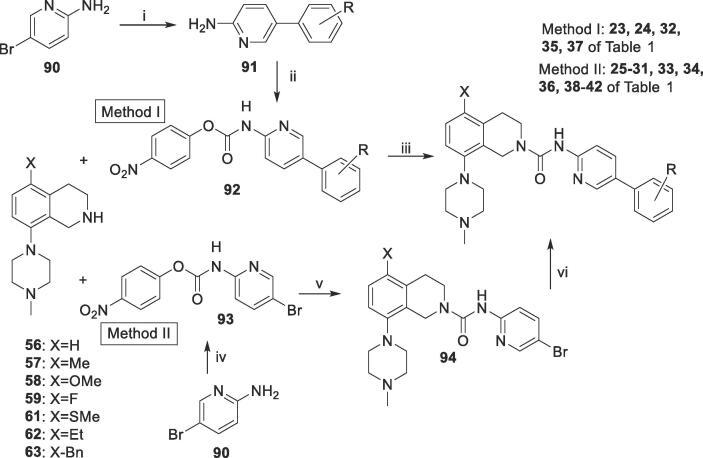
Synthesis of CONH-linked compounds 23–42 of Table 1.

Two general methods, chosen broadly on the availability of the starting materials, were used to prepare the amide-linked series of compounds **23**–**42** of Table 1 (Scheme 4). Reaction of tetrahydroisoquinolines (**56**–**59, 61**–**63**) with preformed nitrobenzylcarbamates **92** gave Table I compounds directly. Alternatively, reaction of the tetrahydroisoquinolines with the intermediate 4-nitrophenyl (5-bromopyridin-2-yl)carbamate) **93** gave the bromides **94**, which were coupled with substituted phenylboronic acids (**73**) or **81**.

*Reagents and condition*s: (i) R-PhB(OH)_2_ (**73**) or **81**, Pd(PPh_3_)_4_, 2 M Na_2_CO_3,_ DME, reflux, 2.5 h; (ii) 4-nitrophenyl chloroformate, pyridine, DCM, 20 °C, 15 h; (iii) MeCN, 70 °C, overnight; (iv) 4-nitrophenylchloroformate, pyridine, DCM, 2–20 °C, overnight (v) DMF, 75 °C, 26 h; (vi) R-PhB(OH)_2_ (**73**) or **81**, PdCl_2_dppf, 2 M Na_2_CO_3_, toluene/EtOH, 85 °C, 15 h.

**Scheme 5:**
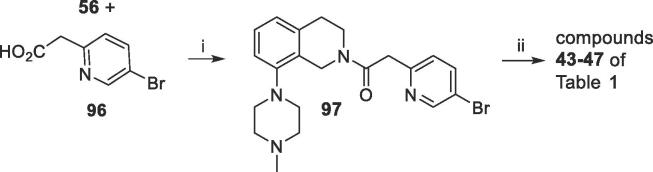
Synthesis of –COCH_2_-linked compounds 43–47 of Table 1.

Compounds **43**–**47** of Table 1 evaluated the utility of a more flexible- COCH_2_– linker. They were prepared as noted in Scheme 5, by reacting tetrahydroisoquinoline **56** with 2-(5-bromopyridin-2-yl)acetic acid **96** to give the bromide **97**. This underwent Suzuki coupling with substituted phenylboronic acids (**73**) or **81** to provide the listed compounds.

*Reagents and conditions*: (i) EDCI, DIPEA, HOBt, DMAP, DMF, 20 °C, 15 h; (ii) R-PhB(OH)_2_ (**73**) or **81**, PdCl_2_dppf, 2 M Na_2_CO_3_, toluene/EtOH, 85 °C, 15 h.

**Scheme 6:**
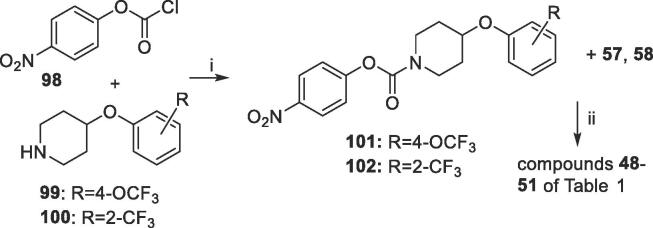
Synthesis of –CO-linked non-aromatic sidechain compounds 48–51 of Table 1.

Compounds **48**–**51** in Table 1, with carbamate-linked non-aromatic sidechain terminal units, were prepared by reacting 4-nitrophenylcarbonyl chloride **98** and amines **99**, **100** to give the 4-nitrophenylcarbamate intermediates **101** and **102**, which were in turn coupled with tetrahydroisoquinolines **57** or **58** (Scheme 6).

*Reagents and conditions*: (i) NEt_3_, DCM, 20 °C, 2 h (90%); (ii) DMAP, toluene, 110 °C, 3 days.

**Scheme 7:**
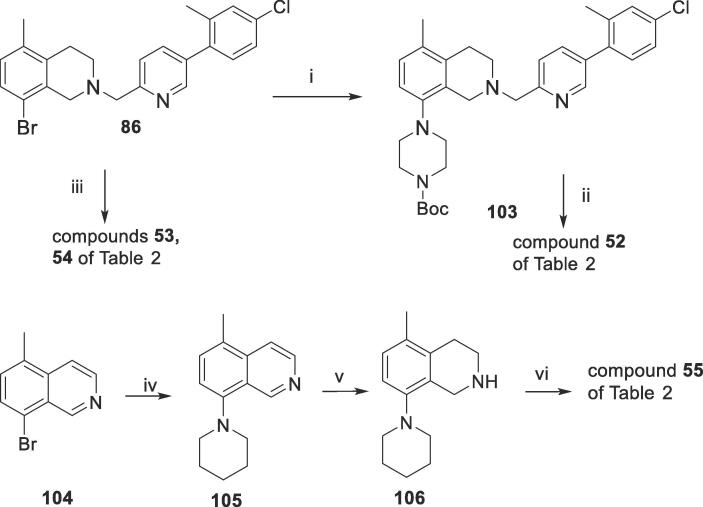
Syntheses of the tetrahydroisoquinolines 52–55 of Table 2.

Finally, the compounds of Table 2 explore variations in the 8-*N*-methylpiperazine unit, by replacing it with a range of related 6-membered heterocycles containing a fixed 2-pyridyl linker and a 2-methyl-4-chlorophenyl side chain (compounds **52**–**55** of Table 2). Similar to the synthesis of compound **12**, treatment of **86** (see Scheme 2B) with various cyclic amines gave compounds **52**–**54**. (Scheme 7). Compound **55** was synthesised by the same procedure as for compound **13** in Table 1 by the *N*-alkylation reaction of **106** with **84**. **106** was prepared from **104** by Buchwald amination followed by reduction.

**Table 2:** Structures of 8-substituted analogues and their *in vitro* assay data.

		MIC (µg/mL)		IC_50_	
No	Z	MABA^a^	LORA^b^	VERO^c^	clogP^d^
**12**		0.79	5.2	14	5.86
**52**		1.8	4.4	12	5.29
**53**		7.8	12	>32	5.30
**54**		3.4	5.9	>32	6.14
**55**		2.6	ND^e^	>32	6.69

^a–d^ As for Table 1; ^e^Not done.

*Reagents and conditions*: (i) Pd_2_(dba)_3_, NaO*^t^*Bu, *rac*-BINAP, toluene, *N*-BOC-piperidine, 100 °C, 5 h; (ii) TFA, DCM; (iii) conditions as for i with morpholine or thiomorpholine; (iv) conditions as for i with piperidine; (v) NaBH_3_CN, MeOH, 0 °C for 10 min, then BF_3_.OEt_2_ 0 °C for 1 h and reflux 3 h; (vi) **84**, K_2_CO_3_, DMF, 20 °C, 5 h.

### 2.2 Structure-activity relationships

The compounds of Table 1 were evaluated for their minimum inhibitory concentrations (MICs; µg/mL) against *Mycobacterium tuberculosis* (strain H37Rv) cultured under both aerobic (MABA)^12^ and anerobic (LORA)^13^ conditions. The mammalian cytotoxicity (IC_50_s) of the compounds was assessed against a VERO cell line,^14^ and the results are recorded in Table 1.

The active compounds possess a wide range of lipophilicities, with calculated clogP values from 2.59 to 7.12. Previously, in several series of quite different classes of compounds that hit different *M.tb* targets, overall positive correlations are seen between antitubercular potency (measured as MICs) and compound lipophilicity (measured as clogP),^15^, ^16^, ^17^ and the same broad trend is shown here for the compounds with measurable MICs (equation 1).

(1) Log(MIC_MABA_) = -0.31(±0.06)clogP + 2.25 (±0.30)

n = 44, R = 0.65, F_1,42_ = 30.1

However, some structure–activity trends over and above this one can be seen. MIC potency falls off sharply at overall clogP values below about 4.5 (e.g., compounds **31, 33, 43**–**47**), regardless of the molecular structure. Due to the diversity of structures in the set of compounds, it is more difficult to identify positive SAR contributions to potency. Compounds (**28**–**30**) with the most lipophilic X substituents Me, Et or Bn had an average MIC (MABA) for of 2.8 µg/mL, compared to an average MIC of 5.0 µg/mL for a larger group of 35 compounds where X was H or OMe, but this may be just the global lipophilicity effect.

For the vast majority of the compounds, the Y-component was a 2-pyridyl, attached to the tetrahydroisoquinoline by a variety of linkers of varying polarity and geometry. Since not all of these sets contained an identical set of R substituents on the terminal phenyl ring there is some noise in the data, but some conclusions can be drawn for the larger sets. Thus compounds **6**–**9** (direct link), **10**–**17** (CH_2_ link), and **23**–**42** (para CONH link) had average MICs (MABA) of 3.3, 4.1 and 3.7 µg/mL respectively, whereas compounds **18**–**22** CO linker) and **43**–**47** (COCH_2_ linker) had much poorer average MICs (12.2 and 17.7 µg/mL respectively). The aliphatic-linked compounds (**48**–**51**) showed potencies in between, with an average MIC of 8.3 µg/mL. Finally, the average MIC (MABA) for the 11 compounds bearing (more lipophilic) alkyl 5-substituents was 2.8 µg/mL, compared with average MICs of 4.8 µg/mL for the 17 5-F compounds and 5.4 µg/mL for the 18 5-OMe compounds of Table 1.

From these results, we selected the CH_2_-pyridyl linker and the 2-methyl-4-chlorophenyl terminal group as suitable for exploring the nature of the substituent space around the tetrahydroisoquinoline 8-position, by replacing the *N*-methylpiperazine with various alternates (Table 2).

The results show that a substituted piperazine functional group at the tetrahydroisoquinoline 4-position provides the best anti-tubercular activity (compounds **12** and **52**). The more lipophilic *N*-piperidyl compound **55** maintains relatively good potency in the MABA assay.

Representative examples of the more potent compounds of Table 1 were evaluated in human and mouse liver microsomes to determine their primary microsomal clearance rates (Table 3). All of the compounds evaluated showed much faster clearance than bedaquiline (**1**), where the very slow human clearance of bedaquiline and its M2 metabolite may contribute to drug-related toxicities.^18^

**Table 3:** Microsomal clearance data for representative compounds of Table 1.

No	HLM Cl_int_^a^	MLM Cl_int_^a^
**1**	3.0	7.0
**6**	22	35
**7**	41	56
**8**	17	42
**10**	121	238
**12**	92	239
**27**	107	810
**36**	48	584
**42**	50	65
**48**	48	40

^a^Clearance (µL/min/kg protein). HLM (Human liver microsomes), MLM (Mouse liver microsomes).

Bedaquiline (**1**) and representative compounds of the tetrahydroisoquinolines of Table 1 were also evaluated for their ability against and selectivity for the mycobacterial (*M. smegmatis)* ATP synthase enzyme (Table 4). Bedaquiline (**1**) is known to be a potent) and very selective inhibitor of mycobacterial ATP synthase,^19^ with an IC_50_ of 0.55 µg/mL for the *M. smegmatis* enzyme. The most potent of the tetrahydroisoquinolines was compound **42** (IC_50_ of 1.8 µg/mL) and a lesser but still useful selectivity (9.4-fold) over the human enzyme. Other analogues (e.g., **10**, **23**) were less potent but more selective inhibitors (>70- and >80-fold respectively).

**Table 4:** ATP synthase inhibition for representative compounds of Table 1.

No	*M. smeg* ATPsynth^a^	Mamm. ATPsynth^a^
**1**	0.55	>1000
**6**	5.5	23
**8**	4.2	16
**10**	7.2	>500
**23**	6.2	>500
**26**	14	13
**27**	2.2	7.2
**35**	6.3	20
**36**	3.0	27
**42**	1.8	17
**49**	6.2	48

^a^IC_50_ values (µg/mL) for inhibition of *M. smegmatis* and human mitochondrial ATP synthase.^22^

## 3 Conclusions

A new class of tetrahydroquinoline compounds are effective inhibitors of *M.tb* in culture, with one-third of the compounds showing MICs of <2 µg/mL. As expected^13-15^ there was a broad general trend of improved potency with higher lipophilicity, with potency falling off sharply below clogP below 4.5. Large substituents (e.g., Bn) at the tetrahydroquinoline 5-position were well-tolerated. There were interesting group differences between sets of compounds with varying linker units Y; compounds **18**–**22** (–CO- linker) and compounds **43**–**47** (–COCH_2_- linker) were much less effective. This might be due to different positioning of the terminal aromatic ring for target binding, which does seem to be important, since compounds **48**–**51**, with a non-aromatic terminal ring, were also relatively ineffective.

Pleasingly, all of the representative compounds evaluated in more detail for microsomal clearance showed much faster rates than **1**, and were effective inhibitors of *M. tb* (*M. smegmatis*) ATP synthase, albeit with lesser differentials than **1** for *M. tb* over human enzyme.

## 4 Experimental

### 4.1 Chemistry

Final products were analysed by reverse-phase HPLC (Alltima C18 5 µm column, 15 × 3.2 mm; Alltech Associated, Inc., Deerfield, IL) using an Agilent HP1100 equipped with a diode-array detector. Mobile phases were gradients of 80% CH_3_CN/20% H_2_O (v/v) in 45 mM NH_4_HCO_2_ at pH 3.5 and 0.5 mL/min. Purity was determined by monitoring at 330 ± 50 nm and was ≥95% for all final products. Melting points were determined on an Electrothermal 9100 melting point apparatus. NMR spectra were obtained on a Bruker Avance 400 spectrometer at 400 MHz for ^1^H. Low-resolution atmospheric pressure chemical ionization (APCI) mass spectra were measured for methanol solutions on a ThermoFinnigan Surveyor MSQ mass spectrometer, connected to a Gilson autosampler.

#### 4.1.1 Preparation of 5-substituted dihydroisoquinoline intermediates **61**–**63** (Scheme 1)

##### 4.1.1.1 8-(4-Methylpiperazin-1-yl)-5-(methylthio)isoquinoline **(61)**

A solution of 4- methylpiperazin-1-yl)isoquinoline (**60**) (1.76 g, 7.73 mmol) in DMF (20 mL) was cooled to 2 °C, *N*-bromosuccinimide (1.4 g, 8.11 mmol) was added, and the mixture was stirred at room temperature for 66 h. The mixture was diluted with ice water and the aqueous layer was extracted with DCM (5x). The combined extract was washed with water, brine, dried (MgSO_4_) and concentrated under reduced pressure. The crude product was purified by flash chromatography eluting with 3–9% MeOH/DCM to yield 5-bromo-8-(4-methylpiperazin-1-yl)isoquinoline (**64**) as a brown solid (1.69 g, 71%); mp 91–93 °C. ^1^H NMR (CDCl_3_) *δ* 9.56 (d, *J* = 0.6 Hz, 1H), 8.62 (d, *J* = 5.9 Hz, 1H), 8.62 (dd, *J* = 5.9, 0.8 Hz, 1H), 7.85 (d, *J* = 8.1 Hz, 1H), 7.01 (d, *J* = 8.1 Hz, 1H), 3.19 (br, 4H), 2.73 (br, 4H), 2.43 (s, 3H). MS (APCI): [M + H]^+^ 306.2 [^79^Br], 308.2 [^81^Br].

A solution of **64** (1.47 g, 4.80 mmol) in anhydrous THF (12 mL) at −78 °C under nitrogen was treated dropwise with *n*-butyllithium (2.6 mL, 5.28 mmol). The mixture turned black and was maintained at −78 °C for 5 min. Methyl disulfide (0.86 mL, 9.6 mmol) was added dropwise under nitrogen and the mixture was stirred at −78 °C for 2 h. The mixture was then quenched with water at −78 °C and then further diluted with water. The aqueous mixture was extracted with EtOAc (2x), and the combined extract was washed with brine, dried (MgSO_4_) and concentrated under reduced pressure to give a dark brown oil. Flash chromatography of the crude product using 4–8% MeOH/DCM as eluent gave 8-(4-methylpiperazin-1-yl)-5-(methylthio)isoquinoline (**65**) as a dark brown oil (95% pure). ^1^H NMR (CDCl_3_) *δ* 9.58 (d, *J* = 0.9 Hz, 1H), 8.58 (d, *J* = 5.9 Hz, 1H), 8.06 (dd, *J* = 5.9, 0.9 Hz, 1H), 7.59 (d, *J* = 8.0 Hz, 1H), 7.10 (d, *J* = 8.0 Hz, 1H), 3.19 (br, 4H), 2.74 (br, 4H), 2.51 (s, 3H), 2.43 (s, 3H). MS (APCI): 274.1 [M + H]^+^.

A solution of **65 (**0.934 g, 3.42 mmol) in MeOH (52 mL) at 2 °C under nitrogen was treated with NaCNBH_3_ (966 mg, 15.37 mmol) in three portions. After stirring at 2 °C for 10 min, BF_3_.OEt_2_ (1.9 mL, 15.37 mmol) was added dropwise. The orange mixture was maintained at the same temperature for another 30 min, then was refluxed under nitrogen for 22 h. The cooled mixture was then neutralised with 2 M Na_2_CO_3_ solution and extracted with EtOAc (3x). The combined organic extract was washed with brine, dried (MgSO_4_) and concentrated under reduced pressure to give a yellow solid. Flash chromatography on silica using 5–33% MeOH/DCM gave **61** as a yellow solid (0.643 g, 68%). mp 77–79 °C. ^1^H NMR (CDCl_3_) *δ* 7.06 (d, *J* = 8.4 Hz, 1H), 6.96 (d, *J* = 8.4 Hz, 1H), 4.00 (s, 2H), 3.17 (t, *J* = 6.2 Hz, 2H), 2.88 (t, *J* = 4.8 Hz, 4H), 2.73 (t, *J* = 6.2 Hz, 2H), 2.55 (br, 4H), 2.43 (s, 3H), 2.35 (s, 3H). MS (APCI): 274.4 [M + H]+

##### 4.1.1.2 5-Ethyl-8-(4-methylpiperazin-1-yl)-1,2,3,4-tetrahydroisoquinoline **(62)**

A solution of **64** (1.49 g, 4.15 mmol) in anhydrous THF (10 mL) at −78 °C under nitrogen was treated with *n*-butyllithium (2.3 mL, 4.56 mmol), then stirred at −78 °C for 5 min. Acetaldehyde (0.70 mL, 12.44 mmol) was added dropwise under nitrogen and the mixture was stirred at −60 °C for 30 min, then gradually warmed up to −10 °C over 30 min. The mixture was quenched with saturated NH_4_Cl solution, diluted with water and extracted with EtOAc (5x). This solution was washed with brine, dried (MgSO_4_) and concentrated under reduced pressure to give the crude product. Flash chromatography using 4–12% MeOH in DCM provided 1-(8-(4-methylpiperazin-1-yl)isoquinolin-5-yl)ethan-1-ol (**66**) as a brown gel (713 mg, contaminated with minor impurities), which was used in the next step without further purification. ^1^H NMR (CDCl_3_) *δ* 9.61 (d, *J* = 0.8 Hz, 1H), 8.54 (d, *J* = 6.0 Hz, 1H), 7.89 (dd, *J* = 6.0, 0.7 Hz, 1H), 7.76 (d, *J* = 7.8 Hz, 1H), 7.14 (d, *J* = 7.8 Hz, 1H), 5.54 (q, *J* = 6.4 Hz, 1H), 3.18 (br, 4H), 2.73 (br, 4H), 2.43 (s, 3H), 1.65 (d, *J* = 6.4 Hz, 3H). MS (APCI): 272.4 [M + H]^+^.

Triethylsilane (4.2 mL, 26.33 mmol) was added slowly to a solution of **66** (0.712 g, 2.63 mmol) in trifluoroacetic acid (30 mL) at room temperature, and then heated at 75 °C for 1 h. The mixture was poured into cold saturated NaHCO_3_ solution and 4 M NaOH solution was added to bring the mixture to pH 14. The aqueous mixture was extracted with DCM (4x), and the combined organic extract was washed with brine, dried (MgSO_4_) and concentrated under reduced pressure to give the crude product as a brown oil. Flash chromatography using 0–10% MeOH/DCM gave 5-ethyl-8-(4-methylpiperazin-1-yl)isoquinoline (**67**) as a dark brown oil (477 mg, 71%). ^1^H NMR (CDCl_3_) *δ* 9.61 (s, 1H), 8.53 (d, *J* = 5.9 Hz, 1H), 7.75 (d, *J* = 5.9 Hz, 1H), 7.43 (d, *J* = 7.7 Hz, 1H), 7.08 (d, *J* = 7.7 Hz, 1H), 3.17 (br, 4H), 3.00 (q, *J* = 7.5 Hz, 2H), 2.72 (br, 4H), 2.42 (s, 3H), 1.34 (t, *J* = 7.5 Hz, 3H). MS (APCI): 256.2 [M + H]^+^.

A solution of **67 (**0.51 g, 2.0 mmol) in MeOH (32 mL) at 2 °C under nitrogen was treated with NaCNBH_3_ (0.564 g, 8.99 mmol) in three portions. The mixture was stirred at the same temperature for 10 min, then BF_3_.OEt_2_ (0.85 mL, 8.987 mmol) was added dropwise. The mixture was kept at 2 °C for 30 min, then heated to reflux under nitrogen overnight. The cooled mixture was neutralised with 2 M Na_2_CO_3_ and extracted with DCM (5x). The combined organic extracts were washed with brine, dried (MgSO_4_) and concentrated under reduced pressure to give a yellow solid. This solid was purified by flash chromatography on silica using 5–15% MeOH/DCM to give **62** as a yellow solid (0.333 g, 64%); mp 72–75 °C. ^1^H NMR (CDCl_3_) *δ* 7.05 (d, *J* = 8.1 Hz, 1H), 6.93 (d, *J* = 8.1 Hz, 1H), 4.04 (s, 2H), 3.17 (t, *J* = 6.2 Hz, 2H), 2.88 (t, *J* = 4.7 Hz, 4H), 2.73 (t, *J* = 6.2 Hz, 2H), 2.52–2.57 (m, 6H), 2.35 (s, 3H), 1.20 (t, *J* = 7.5 Hz, 3H). MS (APCI): 260.2 [M + H]^+^.

##### 4.1.1.3 5-Benzyl-8-(4-methylpiperazin-1-yl)-1,2,3,4-tetrahydroisoquinoline **(63)**

A solution of 5-bromo-8-(4-methylpiperazin-1-yl)isoquinoline (**64**) (0.975 g, 3.13 mmol) in anhydrous THF (8 mL) at −78 °C under nitrogen was treated dropwise with *n*-butyllithium (1.7 mL, 3.4 mmol). The mixture turned dark brown and was maintained at −78 °C for 2 min. Benzaldehyde (0.95 mL, 9.4 mmol) was added dropwise and the mixture was stirred at −78 °C for 2 h, then quenched with half-saturated NH_4_Cl solution and diluted with water. The aqueous mixture was extracted with EtOAc (4x), and the combined extract was washed with brine, dried (MgSO_4_) and concentrated under reduced pressure to give the crude product as a brown oil. Flash chromatography of the crude product (0–12% MeOH in DCM as eluent) provided (8-(4-methylpiperazin-1-yl)isoquinolin-5-yl)(phenyl)methanol (**68**) as a pale yellow semi-solid (568 mg, 55%). ^1^H NMR (CDCl_3_) *δ* 9.58 (d, *J* = 0.7 Hz, 1H), 8.44 (d, *J* = 6.0 Hz, 1H), 7.80 (dd, *J* = 6.0, 0.7 Hz, 1H), 7.68 (d, *J* = 7.8 Hz, 1H), 7.27–7.37 (m, 5H), 7.11 (d, *J* = 7.8 Hz, 1H), 6.41 (s, 1H), 3.17 (br, 4H), 2.73 (br, 4H), 2.43 (s, 3H). MS (APCI): 334.2 [M + H]^+^.

A solution of **68** (0.557 g, 1.67 mmol) in trifluoroacetic acid (18 mL) at room temperature was treated slowly with triethylsilane (2.70 mL, 16.7 mmol). The mixture was heated at 75 °C for 40 min, then left at room temperature overnight. Volatiles were removed under reduced pressure, and the residue was diluted in DCM and neutralised with saturated NaHCO_3_ solution. The aqueous mixture was extracted with DCM (3x), and the combined organic extract was washed with brine, dried (MgSO_4_) and concentrated under reduced pressure to yield the crude product as a brown liquid. The crude product was chromatographed on silica using 0–9% MeOH in DCM to give 5-benzyl-8-(4-methylpiperazin-1-yl)isoquinoline **69** as a yellow brown oil (400 mg, 75%). ^1^H NMR (CDCl_3_) *δ* 9.60 (d, *J* = 0.6 Hz, 1H), 8.47 (d, *J* = 6.0 Hz, 1H), 7.70 (dd, *J* = 6.0, 0.6 Hz, 1H), 7.38 (d, *J* = 7.7 Hz, 1H), 7.28–7.29 (m, 2H), 7.16–7.22 (m, 3H), 7.08 (d, *J* = 7.7 Hz, 1H), 4.34 (s, 2H), 3.19 (br, 4H), 2.73 (br, 4H), 2.43 (s, 3H). MS (APCI): 318.2 [M + H]^+^.

NaCNBH_3_ (0.350 g, 5.57 mmol) was added in three portions to a solution of **69** (0.393 g, 1.24 mmol) in MeOH (19 mL) at 2 °C under nitrogen. The mixture was stirred at 2 °C for 10 min, then BF_3_.OEt_2_ (0.52 mL, 5.6 mmol) was added dropwise. The mixture was maintained at the same temperature for another 30 min and subsequently refluxed under nitrogen for 3 h. The mixture was then cooled to room temperature and then neutralised with 2 M Na_2_CO_3_ solution, then extracted with DCM (3x). The combined organic extract was washed with brine, dried (MgSO_4_) and concentrated under reduced pressure to furnish 5-benzyl-8-(4-methylpiperazin-1-yl)-1,2,3,4-tetrahydroisoquinoline (**63**) as a yellow solid (0.383 g, 96%); mp 116–118 °C. ^1^H NMR (CDCl_3_) *δ* 7.24–7.28 (m, 2H), 7.16–7.20 (m, 1H), 7.11–7.13 (m, 2H), 6.96 (d, *J* = 8.1 Hz, 1H), 6.90 (d, *J* = 8.1 Hz, 1H), 4.02 (s, 2H), 3.90 (s, 2H), 3.10 (t, *J* = 6.2 Hz, 2H), 2.89 (t, *J* = 4.7 Hz, 4H), 2.63 (t, *J* = 6.2 Hz, 2H), 2.55 (m, 4H), 2.35 (s, 3H). MS (APCI): 322.2 [M + H]^+^.

#### 4.1.2 Preparation of the directly-linked 2-pyridyl compounds **6**–**9** (Scheme 2A)

##### 4.1.2.1 N-(5-(4-chloro-2-methylphenyl)pyridin-2-yl)-5-methyl-8-(4-methylpiperazin-1-yl)-3,4-dihydroisoquinoline-2(1H)-carboxamide **(6)**

A mixture of **57** (300 mg, 1.22 mmol) and **70** (434 mg, 1.83 mmol) in toluene (10 mL) was purged with nitrogen before xantphos (35 mg, 0.06 mmol) and Pd_2_(dba)_3_ (28 mg, 0.03 mmol) were added. The mixture was heated in an oil bath at 80 °C, then NaO*^t^*Bu (176 mg, 1.83 mmol) was added. The resulting mixture was heated at 100 °C for 4 h under nitrogen, then it was cooled to room temperature and ice was added to quench the reaction. The aqueous phase was extracted with EtOAc, the combined organic phase was washed with brine, dried over anhydrous Na_2_SO_4_ and filtered through a pad of alumina. The solvent was removed to give the crude product, this was purified by alumina chromatography, eluting with a mixture of EtOAc and petrol ether (1:4) to give 2-(5-bromopyridin-2-yl)-5-methyl-8-(4-methylpiperazin-1-yl)-1,2,3,4-tetrahydroisoquinoline (**71**) as a white solid (407 mg, 83%). ^1^H NMR (CDCl_3_) *δ* 8.23 (d, *J* = 2.4 Hz, 1H), 7.55 (dd, *J* = 9.0, 2.5 Hz, 1H), 7.04 (d, *J* = 8.0 Hz, 1H), 6.90 (d, *J* = 8.0 Hz, 1H), 6.59 (d, *J* = 9.0 Hz, 1H), 4.52 (s, 2H), 3.75 (t, *J* = 5.7 Hz, 2H), 2.96 (t, *J* = 5.7 Hz, 2H), 2.92 (t, *J* = 4.7 Hz, 4H), 2.59 (br, 4H), 2.37 (s, 3H), 2.28 (s, 3H). MS (APCI): 490.2 [^79^Br], 492.2 [^81^Br] [M + H]^+^.

A mixture of **71** (100 mg, 0.25 mmol), (4-chloro-2-methylphenyl)boronic acid (**81**) (127 mg, 0.75 mmol) and aqueous Na_2_CO_3_ (2 M, 0.75 mL, 1.50 mmol) in toluene (2 mL) and EtOH (1 mL) was purged with nitrogen before (dppf)PdCl_2_.DCM (10 mg, 0.012 mmol) was added. The resulting mixture was heated in an oil bath at 85 °C for 24 h. After the solvent was removed, the residue was taken in EtOAc and washed with water and brine, dried over anhydrous Na_2_SO_4_ and filtered through a pad of alumina. The solvent was removed to give the crude product. Chromatography on alumina (4:1 hexanes:EtOAc), followed by trituration with Et_2_O, gave **6** as a white solid (72 mg, 65%); mp 169–172 °C. HPLC 96.5%. ^1^H NMR (CDCl_3_) *δ* 8.17 (d, *J* = 2.3 Hz, 1H), 7.47 (dd, *J* = 8.7, 2.4 Hz, 1H), 7.21 (dd, *J* = 8.2, 2.1 Hz, 1H), 7.13 (d, *J* = 8.2 Hz, 1H), 7.06 (d, *J* = 8.0 Hz, 1H), 6.91 (d, *J* = 8.0 Hz, 1H), 6.75 (d, *J* = 8.8 Hz, 1H), 4.61 (s, 2H), 3.84 (t, *J* = 5.7 Hz, 2H), 3.00 (t, *J* = 5.6 Hz, 2H), 2.93 (t, *J* = 4.7 Hz, 4H), 2.60 (br, 4H), 2.37 (s, 3H), 2.31 (s, 3H), 2.29 (s, 3H). HRMS calcd. for C_27_H_31_ClN_4_ (M + H^+^) *m*/*z* 447.2310, found 447.2296.

##### 4.1.2.2 N-(5-(4-(tert-butyl)phenyl)pyridin-2-yl)-5-methyl-8-(4-methylpiperazin-1-yl)-3,4-dihydroisoquinoline-2(1H)-carboxamide (**7**)

Using the same procedure as for compound **6**, Reaction of **71** with 4-(*tert*-butyl)phenyl)boronic acid (**73**: R = 4-tBu) (133 mg, 0.75 mmol), gave **7** as white crystals (72 mg, 64%); HPLC 99.4%. mp 151–153 °C. ^1^H NMR (CDCl_3_) *δ* 8.50 (d, *J* = 2.3 Hz, 1H), 7.75 (dd, *J* = 8.8, 2.5 Hz, 1H), 7.50–7.44 (m, 4H), 7.05 (d, *J* = 8.0 Hz, 1H), 6.91 (d, *J* = 8.0 Hz, 1H), 6.77 (d, *J* = 8.8 Hz, 1H), 4.61 (s, 2H), 3.84 (t, *J* = 5.7 Hz, 2H), 3.00 (t, *J* = 5.6 Hz, 2H), 2.93 (t, *J* = 4.7 Hz, 4H), 2.59 (br, 4H), 2.37 (s, 3H), 2.31 (s, 3H), 1.36 (s, 9H). HRMS calcd. for C_30_H_38_N_4_ (M + H^+^) *m*/*z* 498.3169, found 498.3153.

##### 4.1.2.3 N-(5-(4-Chloro-2-methylphenyl)pyridin-2-yl)-5-methoxy-8-(4-methylpiperazin-1-yl)-3,4-dihydroisoquinoline-2(1H)-carboxamide **(8)**

To a solution of **74** in aqueous HBr (48% wt, 6 mL) at −5 °C was added bromine (0.25 mL, 9.71 mmol) and aqueous HCl (37% wt, 1.13 mL, 11.3 mmol), followed by dropwise addition of a solution of NaNO_2_ (1.19 g, 17.4 mmol) in water (6 mL). The mixture was stirred at −5 °C for 1 h, then it was neutralized with 1 M NaOH. The aqueous layer was extracted with DCM, dried and evaporated. Column chromatography (49:1 hexanes:EtOAc) afforded 2-bromo-5-(4-chloro-2-methylphenyl)pyridine (**75**) as a white solid (0.36 g, 28%);, which was used directly. ^1^H NMR (CDCl_3_) *δ* 8.32 (dd, *J* = 2.3 Hz, *J* = 0.8 Hz, 1H), 7.55 (dd, *J* = 8.1 Hz, *J* = 0.8 Hz, 1H), 7.48 (dd, *J* = 8.1 Hz, *J* = 2.3 Hz, 1H), 7.30 (d, *J* = 2.2 Hz, 1H), 7.23–7.28 (m, 1H), 7.11 (d, *J* = 8.1 Hz, 1H), 2.24 (s, 3H).

A solution of **75** (0.108 g, 0.383 mmol) in toluene (5 mL) was purged with nitrogen for 1 min. BINAP (0.012 g, 0.019 mmol), Pd_2_(dba)_3_ (0.017 g, 0.019 mmol) and 5-methoxy-8-(4-methylpiperazin-1-yl)-1,2,3,4-tetrahydroisoquinoline (**58**, 0.100 g, 0.383 mmol) were then added, and the mixture stirred at 80 °C for 15 min. A bright orange solution formed, upon which NaO*^t^*Bu (0.055 g, 0.574 mmol) was added, and the mixture heated at reflux for 1 h. The reaction was cooled and partitioned between EtOAc and water, and the organic phase was dried, filtered and evaporated. Column chromatography (19:1 DCM:MeOH) gave **8** as a light brown solid (0.085 g, 48%); mp 62–64 °C. ^1^H NMR (CDCl_3_) *δ* 8.15 (dd, *J* = 2.4 Hz, *J* = 0.6 Hz, 1H), 7.54 (dd, *J* = 8.7 Hz, *J* = 2.4 Hz, 1H), 7.26–7.28 (m, 1H), 7.20 (dd, *J* = 8.1 Hz, *J* = 1.9 Hz, 1H), 7.12 (d, *J* = 8.1 Hz, 1H), 7.03 (d, *J* = 8.7 Hz, 1H), 6.74 (dd, *J* = 8.7 Hz, *J* = 1.5 Hz, 2H), 4.72 (s, 2H), 3.90 (t, *J* = 6.0 Hz, 2H), 3.81 (s, 3H), 2.97 (t, *J* = 4.7 Hz, 4H), 2.91 (t, *J* = 6.0 Hz, 2H), 2.71 (s, 4H), 2.47 (s, 3H), 2.28 (s, 3H). Anal. (C_27_H_31_ClN_4_O) C, H, N.

##### 4.1.2.4 N-(5-(3,5-bis(trifluoromethyl)phenyl)pyridin-2-yl)-5-methoxy-8-(4-methylpiperazin-1-yl)-3,4-dihydroisoquinoline-2(1H)-carboxamide **(9)**

Reaction of **58** (150 mg, 0.57 mmol), **70** (204 mg, 0.86 mmol), xantphos (17 mg, 0.03 mmol) and Pd_2_(dba)_3_ (13 mg, 0.014 mmol) and NaOBu*^t^* (83 mg, 0.86 mmol) in toluene (5 mL) using conditions developed for the synthesis of **71** and purification by alumina chromatography (1:2 EtOAc: hexanes) gave 2-(5-bromopyridin-2-yl)-5-methoxy-8-(4-methylpiperazin-1-yl)-1,2,3,4-tetrahydroisoquinoline (**72**) as a pale solid (200 mg, 83%); ^1^H NMR (CDCl_3_) *δ* 8.21 (dd, *J* = 0.4, 2.5 Hz, 1H), 7.53 (dd, *J* = 9.0, 2.6 Hz, 1H), 7.00 (d, *J* = 8.7 Hz, 1H), 6.72 (d, *J* = 8.7 Hz, 1H), 6.58 (d, *J* = 9.0 Hz, 1H), 4.63 (s, 2H), 3.80 (s, 3H), 3.80 (t, *J* = 6.2 Hz, 2H), 2.91 (t, *J* = 4.7 Hz, 4H), 2.87 (t, *J* = 6.0 Hz, 4H), 2.63 (br, 4H), 2.40 (s, 3H); HRMS calcd. for C_20_H_26_BrN_4_O (M + H^+^) *m*/*z* 417.1285 [^79^Br], 419.1266 [^81^Br], found 417.1270, 419.1253.

A mixture of **72** (80 mg, 0.14 mmol), 3,5-bis(trifluoromethyl)phenyl)boronic acid (**73**: R = 3,5-bisCF_3_) (111 mg, 0.43 mmol) and aqueous Na_2_CO_3_ (2 M, 0.43 mL, 0.86 mmol) in toluene (2 mL) and EtOH (1 mL) was purged with nitrogen before (dppf)PdCl_2_-DCM (6 mg, 0.007 mmol) was added. The resulting mixture was heated in an oil bath at 85 °C for 20 h and then purified using conditions developed for the purification of **6**, to give **9** as a white solid (58 mg, 73%); HPLC 93.7%. mp 145–146 °C. ^1^H NMR *δ* 8.50 (d, *J* = 2.2 Hz, 1H), 7.93 (s, 2H), 7.78–7.74 (m, 2H), 7.03 (d, *J* = 8.8 Hz, 1H), 6.78 (d, *J* = 8.8 Hz, 1H), 6.74 (d, *J* = 8.8 Hz, 1H), 4.77 (s, 2H), 3.91 (t, *J* = 6.1 Hz, 2H), 3.84 (s, 3H), 2.95–2.90 (m, 6H), 2.65 (br, 4H), 2.41 (s, 3H); HRMS calcd. for C_28_H_28_F_6_N_4_O (M + H^+^) *m*/*z* 551.2240, found 551.2227.

**Note**: The X = OMe compounds **8** and **9** were unstable in CDCl_3_ solution over 1–2 days.

#### 4.1.3 Preparation of the –CH_2_-linked 2-pyridyl compounds **10**–**17** of Scheme 2B and Table 1

***General procedure for further purification of methylamine linked compounds of*** Table 1***:*** A minimal amount of EtOH (~1 mL) was added to the tetrahydroisoquinoline (~50 mg) in a vial, then approximately 3 equivalents of 1.25 M methanolic HCl were added. The resulting solution was stirred at room temperature for 5 min, then diisopropyl ether was added dropwise until the solution turned cloudy. The vial was left in the freezer overnight to allow precipitation of the HCl salt. The solution was carefully decanted (using a Pasteur pipette) and the solid carefully rinsed twice with Et_2_O (not filtered open to air, very hydroscopic) and then dried. The solid was then dissolved in water (~5 mL) and added to ~ 50 mL of 1 M aqueous HCl (50 mL). The aqueous layer was extracted twice with Et_2_O (2 × 50 mL), and then aqueous layer was basified with aqueous ammonia. The compound was then back extracted twice with Et_2_O (50 mL) and the organic layer was dried and evaporated to afford pure methylamine linked compounds if Table 1.

##### 4.1.3.1 2-((5-(4-Chlorophenyl)pyridin-2-yl)methyl)-8-(4-methylpiperazin-1-yl)-1,2,3,4-tetrahydroisoquinoline **(10)**

A solution of 8-(4-methylpiperazin-1-yl)-1,2,3,4-tetrahydroisoquinoline (**56**) (0.422 g, 1.82 mmol) and 5-bromo-2-(chloromethyl)pyridine (**76**) (0.414 g, 2.01 mmol) in anhydrous DMF was treated with K_2_CO_3_ (0.378 g, 2.74 mmol) at 20 °C. The mixture was stirred overnight, then diluted with water and extracted with DCM (3x). The combined extract was washed with water, brine, dried (MgSO_4_) and concentrated under reduced pressure. The crude product was chromatographed on silica (0–5% MeOH in DCM) to afford 2-((5-bromopyridin-2-yl)methyl)-8-(4-methylpiperazin-1-yl)-1,2,3,4-tetrahydroisoquinoline (**77**) as a light yellow oil (0.525 g, 72%). ^1^H NMR (CDCl_3_) *δ* 8.63 (dd, *J* = 2.4, 0.6 Hz, 1H), 7.79 (dd, *J* = 8.3, 2.4 Hz, 1H), 7.44 (d, *J* = 8.2 Hz, 1H), 7.13 (t, *J* = 7.8 Hz, 1H), 6.91 (d, *J* = 7.9 Hz, 1H), 6.86 (d, *J* = 7.5 Hz, 1H), 3.82 (s, 2H), 3.69 (s, 2H), 2.93–2.87 (m, 6H), 2.74 (t, *J* = 6.1 Hz, 2H), 2.51 (br, 4H), 2.34 (s, 3H). MS (APCI): 401.1 [^79^Br], 403.1 [^81^Br] [M + H]^+^.

A mixture of **77** (0.267 mmol), 4-chlorophenylboronic acid (**73**: R = 4-Cl**)** (0.293 mmol) and 2 M Na_2_CO_3_ solution (1.067 mmol) in toluene and EtOH (2:1) was purged with nitrogen. PdCl_2_dppf (5 mol%) was added and the mixture was purged again with nitrogen and heated at 80 °C for 2 h. The cooled mixture was diluted with water and extracted with DCM (3x). The combined organic extracts were washed with brine, dried (MgSO_4_) and evaporated to give the crude product. Chromatography on silica gel, eluting with MeOH/DCM mixtures, gave **10**; mp 140–142 °C. HPLC 97.5%. ^1^H NMR (CDCl_3_) *δ* 8.77 (dd, *J* = 2.3, 0.6 Hz, 1H), 7.83 (dd, *J* = 8.1, 2.4 Hz, 1H), 7.59 (d, *J* = 8.1 Hz, 1H), 7.53 (AB br d, *J* = 8.7 Hz, 2H), 7.45 (AB br d, *J* = 8.7 Hz, 2H), 7.13 (t, *J* = 7.8 Hz, 1H), 6.91 (d, *J* = 7.9 Hz, 1H), 6.89 (d, *J* = 7.9 Hz, 1H), 3.91 (s, 2H), 3.74 (s, 2H), 2.96–2.88 (m, 6H), 2.78 (t, *J* = 6.1 Hz, 2H), 2.50 (br, 4H), 2.32 (s, 3H). HRMS (ESI^+^): [M + H]^+^ calculated for C_26_H_30_ClN_4_: 433.2154, found: 433.2146.

##### 4.1.3.2 2-((5-(2,4-Dichlorophenyl)pyridin-2-yl)methyl)-8-(4-methylpiperazin-1-yl)-1,2,3,4-tetrahydroisoquinoline **(11)**

Similar reaction of **77** and 2-methyl-4-chlorophenylboronic acid (**81**) gave **11** as a brown gel. HPLC 95.5%. ^1^H NMR (CDCl_3_) *δ* 8.52 (dd, *J* = 2.1, 0.9 Hz, 1H), 7.62–7.56 (m, 2H), 7.30 (d, *J* = 2.1 Hz, 1H), 7.28–7.24 (m, 2H), 7.17–7.12 (m, 2H), 6.91 (d, *J* = 7.9 Hz, 1H), 6.88 (d, *J* = 7.9 Hz, 1H), 3.92 (s, 2H), 3.73 (s, 2H), 2.96 (t, *J* = 6.0 Hz, 2H), 2.89 (t, *J* = 4.7 Hz, 2H), 2.82 (t, *J* = 6.0 Hz, 2H), 2.50 (br, 4H), 2.32 (s, 3H), 2.27 (s, 3H). HRMS (ESI^+^): [M + H]^+^ calculated for C_27_H_32_ClN_4_: 447.2310, found: 447.2293.

##### 4.1.3.3 2-((5-(4-Chlorophenyl)pyridin-2-yl)methyl)-8-(4-methylpiperazin-1-yl)-5-methyl-1,2,3,4-tetrahydroisoquinoline **(12**)

A mixture of 5-bromopicolinaldehyde (**80**) (5.00 g, 26.9 mmol), 2-methyl-4-chlorophenyl boronic acid (**81**) (4.16 g, 24.4 mmol) and K_3_PO_4_·H_2_O (11.23 g, 48.8 mmol) in water (25 mL), acetonitrile (75 mL) and dioxane (75 mL) was purged with nitrogen for 1 min. Pd(PPh_3_)_4_ (0.62 g, 0.54 mmol) was then added, and the mixture stirred at reflux for 5 h. The reaction was cooled and the solvent removed under reduced pressure. The residue was partitioned between EtOAc and water, and the organic phase was dried, filtered and evaporated. Column chromatography (19:1, x4:EtOAc) afforded 5-(4-chloro-2-methylphenyl)picolinaldehyde (**82**) as a tan solid (3.20 g, 57%), which was used directly. ^1^H NMR (CDCl_3_) *δ* 10.14 (s, 1H), 8.74 (s, 1H), 8.04 (d, *J* = 7.9 Hz, 1H), 7.82 (d, *J* = 7.9 Hz, 1H), 7.26–7.34 (m, 2H), 7.17 (d, *J* = 8.1 Hz, 1H), 2.28 (s, 3H).

A mixture of **82** (0.91 g, 3.93 mmol) and NaBH_4_ (0.30 g, 7.86 mmol) in MeOH (50 mL, anhydrous) was stirred at 20 °C for 1 h. The solvent was then removed and the residue partitioned between EtOAc and water. The organic layer was dried and evaporated to afford (5-(4-chloro-2-methylphenyl)pyridin-2-yl)methanol (**83**) as a white solid (0.90 g, 98%). ^1^H NMR (CDCl_3_) *δ* 8.50 (d, *J* = 1.6 Hz, 1H), 7.62 (dd, *J* = 8.0 Hz, *J* = 2.2 Hz, 1H), 7.24–7.33 (m, 3H), 7.13 (d, *J* = 8.0 Hz, 1H), 4.83 (s, 2H), 2.25 (s , 3H), which was used directly.

To a solution of **83** (0.26 g, 1.11 mmol) and triethylamine (0.23 mL, 1.67 mmol) in DCM (7.5 mL) at 20 °C was added mesyl chloride (0.10 mL, 1.34 mmol) dropwise. After 15 min, the reaction was diluted with DCM (20 mL) and the organic layer washed with sat. NaHCO_3_, dried and evaporated. The residue was redissolved in acetone (15 mL, anhydrous), lithium bromide (~2.5 g, excess) was added, and the mixture heated at reflux for 1.5 h. The solution was then cooled and the solvent evaporated to give a residue which was partitioned between EtOAc and water. The aqueous layer was extracted twice with EtOAc and the organic layer was dried and evaporated to afford 2-(bromomethyl)-5-(4-chloro-2-methylphenyl)pyridine (**84**) as a light brown solid (0.31 g, 94%), which was used directly. ^1^H NMR (CDCl_3_) *δ* 8.52 (d, *J* = 2.2 Hz, 1H), 7.64 (dd, *J* = 8.0 Hz, *J* = 2.2 Hz, 1H), 7.51 (dd, *J* = 8.0 Hz, *J* = 0.7 Hz, 1H), 7.23–7.33 (m, 3H), 7.13 (d, *J* = 8.0 Hz, 1H), 4.61 (s, 2H), 2.26 (s , 3H).

To a solution of 8-bromo-5-methyl-1,2,3,4-tetrahydroisoquinoline (**85**)^20^ (0.062 g, 0.274 mmol) and **84** (0.105 g, 0.356 mmol) in DMF (2.5 mL) was added K_2_CO_3_ (0.057 g, 0.411 mmol) and the resultant mixture stirred at room temperature for 5 h. The reaction was then diluted with EtOAc (50 mL) and washed with water (10 mL), and the organic layer was dried and evaporated. Column chromatography (4:1, X4:EtOAc) afforded **86** as a yellow oil (0.098 g, 81%). ^1^H NMR (CDCl_3_) *δ* 8.53 (dd, *J* = 2.2 Hz, *J* = 0.7 Hz, 1H), 7.61 (dd, *J* = 8.0 Hz, *J* = 2.2 Hz, 1H), 7.55 (d, *J* = 7.8 Hz, 1H), 7.23–7.33 (m, 3H), 7.17 (d, *J* = 8.0 Hz, 1H), 6.90 (d, *J* = 8.0 Hz, 1H), 3.95 (s, 2H), 3.72 (s, 2H), 2.84 (t, *J* = 5.3 Hz, 2H), 2.78 (t, *J* = 5.3 Hz, 2H), 2.28 (s, 3H), 2.19 (s, 3H), which was used directly.

A solution of **86** (0.100 g, 0.226 mmol) in toluene (2.5 mL) was purged with nitrogen for 1 min. BINAP (0.007 g, 0.011 mmol), Pd_2_(dba)_3_ (0.005 g, 0.0057 mmol) and *N*-methylpiperazine (0.038 mL, 0.34 mmol) were then added, and the mixture stirred at 80 °C for 15 min. A bright orange solution formed, upon which NaO*^t^*Bu (0.105 g, 1.09 mmol) was added, and the mixture stirred at reflux under nitrogen for 4 h. The reaction was cooled and partitioned between EtOAc and water, and the organic phase was dried, filtered and evaporated. Column chromatography (19:1, DCM:MeOH) gave **12** as a yellow oil (0.037 g, 36%), which was further purified by the general salt formation/back extraction procedure as described above. ^1^H NMR (CDCl_3_) *δ* 8.52 (t, *J* = 1.0 Hz, 1H), 7.55–7.62 (m, 2H), 7.23–7.33 (m, 2H), 7.15 (d, *J* = 8.0 Hz, 1H), 7.01 (d, *J* = 8.0 Hz, 1H), 6.88 (d, *J* = 8.0 Hz, 1H), 3.92 (s, 2H), 3.75 (s, 2H), 2.87–2.96 (m, 8H), 2.57 (s, 4H), 2.39 (s, 3H), 2.29 (s, 3H), 2.19 (s, 3H); Anal. (C_28_H_36_Cl_4_N_4_) C, H, N (tri-HCl salt).

##### 4.1.3.4 2-((5-(4-Chlorophenyl)pyridin-2-yl)methyl)-8-(4-methoxypiperazin-1-yl)-5-methyl-1,2,3,4-tetrahydroisoquinoline **(13**)

To a solution of **58** (0.017 g, 0.065 mmol) and **84** (0.023 g, 0.078 mmol) in DMF (1 mL) was added K_2_CO_3_ (0.013 g, 0.098 mmol) and the resultant mixture stirred at r.t. for 5 h. The reaction was then diluted with EtOAc (50 mL) and washed with water (5 × 10 mL), and the organic layer was dried and evaporated. Column chromatography (9:1, DCM:MeOH) afforded **13** as a pale yellow oil (0.025 g, 80%), which was further purified by the general salt formation/back extraction procedure as described above: ^1^H NMR (CDCl_3_) *δ* 8.52 (s, 1H), 7.57–7.62 (m, 2H), 7.23–7.32 (m, 2H), 7.16 (d, *J* = 8.6 Hz, 1H), 6.94 (d, *J* = 8.1 Hz, 1H), 6.68 (d, *J* = 8.6 Hz, 1H), 3.91 (s, 2H), 3.80 (s, 3H), 3.75 (s, 2H), 2.90 (s, 4H), 2.81 (s, 3H), 2.59 (s, 4H), 2.39 (s, 3H), 2.27 (s, 3H); Anal. (C_18_H_14_N_2_O_3_) C, H, N.

##### 4.1.3.5 2-((5-(4-Chloro-2-methylphenyl)pyridin-2-yl)methyl)-8-(4-methylpiperazin-1-yl)-5-(methylthio)-1,2,3,4-tetrahydroisoquinoline **(14)**

Similar reaction of 8-(4-methylpiperazin-1-yl)-5-(methylthio)-1,2,3,4-tetrahydroisoquinoline (**61)** with **76**, and chromatography on silica, eluting with 2–10% MeOH in DCM gave 2-((5-bromopyridin-2-yl)methyl)-8-(4-methylpiperazin-1-yl)-5-(methylthio)-1,2,3,4-tetrahydroisoquinoline (**79**) as a yellow gum (85%). ^1^H NMR (CDCl_3_) *δ* 8.64 (d, *J* = 1.9 Hz, 1H), 7.79 (dd, *J* = 8.3, 2.4 Hz, 1H), 7.43 (d, *J* = 8.3 Hz, 1H), 7.06 (d, *J* = 8.4 Hz, 1H), 6.96 (d, *J* = 8.4 Hz, 1H), 3.82 (2, 2H), 3.70 (s, 2H), 2.88–2.82 (m, 6H), 2.79–2.74 (m, 2H), 2.51 (br, 4H), 2.42 (s, 3H), 2.34 (s, 3H). MS (APCI): 447.1 [^79^Br], 449.1 [^81^Br] [M + H]^+^.

Reaction of **79** with boronic acid (**81**) as above gave **14** (76% yield); mp 65–68 °C. ^1^H NMR (CDCl_3_) *δ* 8.52 (dd, *J* = 2.2, 0.9 Hz, 1H), 7.62–7.55 (m, 2H), 7.30 (d, *J* = 2.1 Hz, 1H), 7.24 (d, *J* = 2.2 Hz, 1H), 7.16 (d, *J* = 8.2 Hz, 1H), 7.06 (d, *J* = 8.4 Hz, 1H), 6.96 (d, *J* = 8.4 Hz, 1H), 3.91 (s, 2H), 3.74 (s, 2H), 2.90–2.85 (m, 8H), 2.50 (br, 4H), 2.42 (s, 3H), 2.32 (s, 3H), 2.27 (s, 3H). HRMS (ESI^+^): [M + H]^+^ calculated for C_28_H_34_ClN_4_S: 493.2117, found: 493.2191

##### 4.1.3.6 2-((5-(4-Chloro-2-methylphenyl)pyridin-2-yl)methyl)-8-(4-methylpiperazin-1-yl)-5-fluoro-1,2,3,4-tetrahydroisoquinoline **(15)**

Similar reaction of 5-fluoro-8-(4-methylpiperazin-1-yl)-1,2,3,4-tetrahydroisoquinoline (**59)** with **76** gave 2-((5-bromopyridin-2-yl)methyl)-8-(4-methylpiperazin-1-yl)-5-fluoro-1,2,3,4-tetrahydroisoquinoline (**78**) (0.237 g, 64%); mp 93–95 °C. ^1^H NMR (CDCl_3_): *δ* 8.64 (d, *J* = 1.9 Hz, 1H), 7.80 (dd, *J* = 8.3, 2.4 Hz, 1H), 7.42 (d, *J* = 8.3 Hz, 1H), 6.91–6.82 (m, 2H), 3.82 (2, 2H), 3.69 (s, 2H), 2.86–2.82 (m, 6H), 2.74 (t, *J* = 6.0 Hz, 2H), 2.50 (br, 4H), 2.33 (s, 3H). MS (APCI): 419.1 [^79^Br], 421.1 [^81^Br] [M + H]^+^.

Reaction of **78** with boronic acid (**81**) as above gave **15** as an oil. HPLC 83.7%. ^1^H NMR (CDCl_3_) *δ* 8.54 (d, *J* = 1.6 Hz, 1H), 7.64 (dd, *J* = 7.9, 2.2 Hz, 1H), 7.57 (d, *J* = 7.9 Hz, 1H), 7.32 (d, *J* = 2.0 Hz, 1H), 7.27 (dd, *J* = 7.9, 1.9 Hz, 1H), 7.18 (d, *J* = 8.2 Hz, 1H), 6.93–6.84 (m, 2H), 3.93 (s, 2H), 3.74 (s, 2H), 2.92–2.82 (m, 8H), 2.50 (br, 4H), 2.33 (s, 3H), 2.28 (s, 3H). HRMS (ESI^+^): [M + H]^+^ calculated for C_27_H_30_ClFN_4_: 465.22158, found: 465.22036.

##### 4.1.3.7 2-((5-(2,4-Difluorophenyl)pyridin-2-yl)methyl)-5-fluoro-8-(4-methylpiperazin-1-yl)-1,2,3,4-tetrahydroisoquinoline **(16)**

Reaction of **78** with boronic acid **73** (R = 2,4-diF) as above gave **16** as an oil. HPLC 78.5%. ^1^H NMR (CDCl_3_) *δ* 8.71 (s, 1H), 7.83 (dt, *J* = 8.1, 1.9 Hz, 1H), 7.58 (d, *J* = 8.1 Hz, 1H), 7.48–7.40(m, 1H), 7.03–6.93 (m, 2H), 6.92–6.82 (m, 2H), 3.92 (s, 2H), 3.73 (s, 2H), 2.89–2.79 (m, 8H), 2.49 (br, 4H), 2.32 (s, 3H). HRMS (ESI^+^): [M + H]^+^ calculated for C_26_H_28_F_3_N_4_: 453.22606, found: 453.22619.

##### 4.1.3.8 2-((5-(4-Chloro-2-(trifluoromethyl)phenyl)pyridin-2-yl)methyl)-5-fluoro-8-(4-methylpiperazin-1-yl)-1,2,3,4-tetrahydroisoquinoline **(17)**

Reaction of **78** with boronic acid **73** (R = 2-CF_3_, 4-Cl) as above gave **17** as a solid (56%); mp 102–104 °C. HPLC 93.9%. ^1^H NMR (CDCl_3_) *δ* 8.81 (d, *J* = 2.2 Hz, 1H), 7.88 (dd, *J* = 8.1, 2.4 Hz, 1H), 7.80 (d, *J* = 8.2 Hz, 1H), 7.73 (s, 1H), 7.63 (d, *J* = 8.2 Hz, 1H), 7.58 (d, *J* = 8.2 Hz, 1H), 6.93–6.83 (m, 2H), 3.94 (s, 2H), 3.75 (s, 2H), 2.88–2.83 (m, 6H), 2.79 (d, *J* = 5.7 Hz, 2H), 2.49 (br, 4H), 2.32 (s, 3H). HRMS (ESI^+^): [M + H]^+^ calculated for C_27_H_28_ClF_4_N_4_: 519.19331, found: 519.19372.

#### 4.1.4 Preparation of the –CO-linked pyridyl side compounds **18**–**22** of Scheme 3 and Table 1

General procedure: A suspension of the substituted phenylpicolinic acids (**87**–**89**) (0.319 mmol) in anhydrous DMF was treated at room temperature under nitrogen with diisopropylethylamine (0.319 mmol). The mixture was then treated with HATU (0.319 mmol) and was stirred for 5 min. Tetrahydroisoquinolines **56** or **58** (0.290 mmol) were added and the mixture was stirred at room temperature overnight. The mixture was diluted in water and the aqueous mixture was extracted with DCM (3x). The combined organic extract was washed with brine, dried (MgSO_4_) and concentrated under reduced pressure to furnish the crude product, which was chromatographed on silica using mixtures of MeOH and DCM to yield the clean products as yellow brown solids in 25–35% yields.

##### 4.1.4.1 (5-(4-Chloro-2-methylphenyl)pyridin-2-yl)(8-(4-methylpiperazin-1-yl)-3,4-dihydroisoquinolin-2(1H)-yl)methanone **(18)**

m.p. 132–135 °C. HPLC 96.0%. ^1^H NMR (CDCl_3_) δ (two rotamers, data on the major one is presented) 8.54 (s, 1H), 7.78–7.69 (m, 2H), 7.30–7.25 (m, 2H), 7.20–7.12 (m, 2H), 7.00–6.93 (m, 2H), 4.90 (s, 2H), 3.85 (br, 2H), 3.40 (br, 4H), 3.20 (br, 4H), 3.00 (br, 7H), 2.25 (br, 3H). HRMS (ESI^+^): [M + H]^+^ calculated for C_27_H_30_ClN_4_O: 461.2103, found 461.2099.

##### 4.1.4.2 (5-(4-Chlorophenyl)pyridin-2-yl)(8-(4-methylpiperazin-1-yl)-3,4-dihydroisoquinolin-2(1H)-yl)methanone **(19)**

m.p. 144–147 °C. HPLC 97.7%. ^1^H NMR (CDCl_3_) δ (two rotamers, data on the major one is presented) 8.78 (d, *J* = 1.8 Hz, 1H), 7.95 (dd, *J* = 8.1, 1.8 Hz, 1H), 7.72 (d, *J* = 8.1 Hz, 1H), 7.58–7.45 (m, 4H), 7.18 (t, *J* = 7.7 Hz, 1H), 7.01–6.97 (m, 2H), 4.89 (s, 2H), 3.80 (t, *J* = 5.6 Hz, 2H), 3.34 (br, 4H), 3.17 (br, 4H), 2.97 (t, *J* = 5.6 Hz, 2H), 2.91 (s, 3H). HRMS (ESI^+^): [M + H]^+^ calculated for C_26_H_28_ClN_4_O: 447.1946, found: 447.1934.

##### 4.1.4.3 (5-(3-Chlorophenyl)pyridin-2-yl)(8-(4-methylpiperazin-1-yl)-3,4-dihydroisoquinolin-2(1H)-yl)methanone **(20)**

m.p. 105–108 °C. HPLC 96.7%. ^1^H NMR (CDCl_3_) δ (two rotamers, data on the major one is presented) 8.81 (d, *J* = 1.8 Hz, 1H), 7.99 (dd, *J* = 8.2, 2.2 Hz, 1H), 7.76 (d, *J* = 8.2 Hz, 1H), 7.53–7.41 (m, 4H), 7.24–7.17 (m, 1H), 7.05 (t, *J* = 7.8 Hz, 1H), 7.02–6.96 (m, 1H), 4.91 (s, 2H), 3.84 (t, *J* = 5.9 Hz, 2H), 3.31 (br, 4H), 3.21 (br, 4H), 3.00 (t, *J* = 5.9 Hz, 2H), 2.90 (s, 3H). HRMS (ESI^+^): [M + H]^+^ calculated for C_26_H_28_ClN_4_O: 447.1946, found: 447.1931.

##### 4.1.4.4 (5-(4-Chlorophenyl)pyridin-2-yl)(5-methoxy-8-(4-methylpiperazin-1-yl)-3,4-dihydroisoquinolin-2(1H)-yl)methanone **(21)**

21% yield; m.p. 124–127 °C. HPLC 99.8%. ^1^H NMR (CDCl_3_) δ (two rotamers, data on the major one is presented) 8.81 (apparent bd,1H), 7.97 (dd, *J* = 8.1, 2.1 Hz, 1H), 7.75 (d, *J* = 8.1 Hz, 1H), 7.55 (AB d, *J* = 8.1 Hz, 1H), 7.49 (AB d, *J* = 8.1 Hz, 1H), 7.06 (d, *J* = 7.7 Hz, 1H), 6.74 (d, *J* = 7.7 Hz, 1H), 4.92 (s, 2H), 3.99 (t, *J* = 5.6 Hz, 2H), 3.81 (s, 3H). 3.09 (br, 6H), 2.94–2.87 (m, 2H), 2.78 (br, 2H), 2.74 (s, 3H). HRMS (ESI + ): [M + H] + calculated for C_27_H_30_ClN_4_O_2_: 477.2052, found: 477.2031.

##### 4.1.4.5 (5-(4-Chloro-2-methylphenyl)pyridin-2-yl)(5-methoxy-8-(4-methylpiperazin-1-yl)-3,4-dihydroisoquinolin-2(1H)-yl)methanone **(22)**

29% yield; mp 125–128 °C. HPLC 98.5%. ^1^H NMR (CDCl_3_) δ (two rotamers, data on the major one is presented) 8.81 (apparent t,1H), 7.77–7.71 (m, 2H), 7.33 (apparent d,1H, 1H), 7.28 (dd, *J* = 8.2, 2.0 Hz, 1H), 7.17 (d, *J* = 8.1 Hz, 1H), 7.08 (d, *J* = 8.7 Hz, 1H), 6.76 (d, *J* = 8.7 Hz, 1H), 4.91 (s, 2H), 3.85–3.82 (m, 5H). 3.26 (br, 2H), 3.16 (br, 4H), 2.96–2.80 (m, 7H), 2.28 (s, 3H). HRMS (ESI^+^): [M + H]^+^ calculated for C_28_H_32_ClN_4_O_2_: 491.2208, found: 491.2202.

#### 4.1.5 Preparation of the –CONH-linked pyridyl side compounds **23**–**42** of Scheme 4 and Table 1

Two general methods were used to prepare this class of compounds, the chosen method was dependant on the availability of the starting materials.

Method 1

##### 4.1.5.1 N-(5-(4-Chloro-2-methylphenyl)pyridin-2-yl)-8-(4-methylpiperazin-1-yl)-3,4-dihydroisoquinoline-2(1H)-carboxamide **(24)**.

A mixture of 2-amino-5-bromopyridine (**90**) (0.996 g, 5.76 mmol), 4-chloro-2-methylphenyl boronic acid (**81**) (0.771 g, 4.53 mmol) and 1 M Na_2_CO_3_ solution (9.5 mL, 9.465 mmol) in dimethoxyethane (18 mL) was flushed with nitrogen for 5 min. Tetrakis(triphenylphosphine)palladium(0) (47.6 mg, 0.0412 mmol) was added. The mixture was flushed again with nitrogen for 5 min, and then refluxed under nitrogen for 2.5 h. The mixture was cooled to room temperature and was diluted in water. The aqueous mixture was extracted with EtOAc (3x). The combined organic extract was washed with brine, dried (MgSO_4_) and concentrated under reduced pressure to furnish the crude product as a brown semi-solid. The crude product was chromatographed on silica using mixtures of hexane/EtOAc and finally 100% EtOAc to afford 4-nitrophenyl-(5-(4-chloro-2-methylphenyl)pyridine-2-yl)carbamate (**91**: R = 2-Me, 4-Cl) as a cream solid (0.905 g, 72%). ^1^HNMR (CDCl_3_) *δ* 8.01 (dd, *J* = 2.4, 0.6 Hz, 1H), *δ* 7.38 (dd, *J* = 8.4, 2.4 Hz, 1H), 7.26–7.25 (partially overlapped with residue of CHCl_3_, 1H), 7.20 (dd, *J* = 8.2, 2.0 Hz, 1H), 7.11 (d, *J* = 8.2 Hz, 1H), 6.56 (dd, *J* = 8.4, 0.7 Hz, 1H), 4.49 (br s, 2H, NH_2_), 2.25 (s, 3H). It was used directly.

Compound **91** (0.392 g, 1.793 mmol) was dissolved in anhydrous DCM (6 mL), anhydrous pyridine (0.17 mL, 2.151 mmol) was added, followed by 4-nitrophenyl chloroformate (443.6 mg, 2.151 mmol). The white slurry was stirred at room temperature under nitrogen overnight. The mixture was passed through a filter paper-lined Buchner funnel under vacuum, the white solid was washed with DCM and further dried to yield *N*-((5-(4-chloro-2-methylphenyl)pyridin-2-yl)carbamoyl)-2-(4-nitrophenyl)acetamide (**92**: R = 4-Cl, 2-Me) as a white solid (0.522 g, 76%); mp 201–203 °C, which was used directly. ^1^H NMR (DMSO‑*d*_6_) δ (3:1 mixture of rotamers) 11.05 (s, 1H), 10.8–10.4 (br s, 1H), 8.29 (t, *J =* 1.5 Hz, 1H), 8.14–8.09 (m, 2H), 7.86 (dd, *J =* 2.5, 0.6 Hz, 1H), 7.85–7.79 (br s, 2H), 7.43 (d, *J =* 2 Hz, 1H), 7.40–7.32 (m, 3H), 7.28 (d, *J =* 8.2 Hz, 1H), 7.28–7.24 (m, 1H), 6.96–6.90 (m, 2H), 2.27 (s, 3H), 2.24 (s, 3H), MS (APCI): 277.2 [^35^Cl] and 279.2 [^37^Cl] [M−*p*−NO_2_PhO + MeO]^+^.

A mixture of **56** (121 mg, 0.52 mmol) and **92** (R = 2-Me, 4-Cl, 301 mg, 0.78 mmol) in acetonitrile (3 mL) was stirred overnight at 70 °C. The mixture was directly adsorbed onto silica and chromatographed using 3–10% MeOH in DCM to afford **24** as pale yellow solid (57%); mp 86–88 °C. HPLC 96.7%. ^1^H NMR (CDCl_3_) *δ* 8.16–8.11 (m, 2H), 7.60 (d, *J* = 8.6, 2.4 Hz, 1H), 7.34 (s, 1H), 7.28 (d, *J* = 2.0 Hz, 1H), 7.24–7.21 (m, 2H), 7.13 (d, *J* = 8.1 Hz, 1H), 7.04 (d, *J* = 7.6 Hz, 1H), 6.96 (d, *J* = 7.4 Hz, 1H), 4.73 (s, 2H), 3.73 (t, *J* = 6.1 Hz, 2H), 2.99–2.94 (m, 6H), 2.66 (br, 4H), 2.39 (s, 3H), 2.26 (s, 3H). HRMS (ESI^+^): [M + H]^+^ calculated for C_27_H_31_ClN_5_O 476.2212, found: 476.2199.

The following compounds were similarly prepared:

##### 4.1.5.2 8-(4-Methylpiperazin-1-yl)-N-(5-(4-(trifluoromethoxy)phenyl)pyridin-2-yl)-3,4-dihydroisoquinoline-2(1H)-carboxamide **(23)**

Intermediate **92** (R = 4-OCF_3_)^21^ was made by the above procedure and used without purification. Reaction of **56** and **92** (R = 4-OCF_3_) as above gave **23** (75% yield); mp 209–211 °C. HPLC 99.7%. ^1^H NMR (CDCl_3_) *δ* 8.42 (dd, *J* = 2.4, 0.6 Hz, 1H), 8.16 (dd, *J* = 8.7, 0.6 Hz, 1H), 7.85 (dd, *J* = 8.7, 2.4 Hz, 1H), 7.58–7.55 (m, 2H), 7.35 (s, 1H), 7.30 (d, *J* = 8.0 Hz, 2H), 7.22 (t, *J* = 7.8 Hz, 1H), 7.04 (d, *J* = 7.6 Hz, 1H), 6.96 (d, *J* = 7.4 Hz, 1H), 4.73 (s, 2H), 3.73 (t, *J* = 6.1 Hz, 2H), 3.00–2.94 (m, 6H), 2.66 (br, 4H), 2.40 (s, 3H). HRMS (ESI^+^): [M + H]^+^ calculated for C_27_H_29_F_3_N_5_O_2_ 512.2268, found: 512.2253.

##### 4.1.5.3 5-Methoxy-N-(6′-methoxy-[3,3′-bipyridin]-6-yl)-8-(4-methylpiperazin-1-yl)-3,4-dihydroisoquinoline-2(1H)-carboxamide **(32)**

Reaction of **58** and **92** (R = 3-aza, 4-OMe) as above gave **32**; mp 93–96 °C. HPLC 95.0%. ^1^H NMR (CDCl_3_) *δ* 8.38 (dd, *J* = 2.4, 0.4 Hz, 1H), 8.35 (dd, *J* = 1.6, 0.6 Hz, 1H), 8.14 (dd, *J* = 8.7, 0.6 Hz, 1H), 7.80 (dd, *J* = 8.7, 2.5 Hz, 1H), 7.74 (dd, *J* = 8.6, 2.6 Hz, 1H), 7.42 (s, 1H, NH), 7.04 (d, *J* = 8.7 Hz, 1H), 6.83 (dd, *J* = 7.6, 0.6 Hz, 1H), 6.75 (d, *J* = 8.7 Hz, 1H), 4.72 (s, 2H), 3.98 (s, 3H), 3.81 (s, 3H), 3.73 (t, *J* = 6.1 Hz, 2H), 2.92–2.88 m, 6H), 2.67 (br, 4H), 2.41 (s, 3H). HRMS (ESI^+^): [M + H]^+^ calculated for C_27_H_33_N_6_O_3_ 489.2609, found: 489.2607.

##### 4.1.5.4 N-(5-(4-chloro-2-methylphenyl)pyridin-2-yl)-5-methoxy-8-(4-methylpiperazin-1-yl)-3,4-dihydroisoquinoline-2(1H)-carboxamide **(35)**

From **58** and **92** (R = 2-Me, 4-Cl). 78% yield; mp 99–101 °C. HPLC 99.5%. ^1^H NMR (CDCl_3_) *δ* 8.14 (dd, *J* = 2.3, 0.7 Hz, 1H), 8.12 (dd, *J* = 8.6, 0.7 Hz, 1H), 7.60 (dd, *J* = 8.6, 2.4 Hz, 1H), 7.41 (s, 1H, NH), 7.28 (d, *J* = 2.1 Hz, 1H), 7.23 (dd, *J* = 8.2, 1.9 Hz, 1H), 7.13 (d, *J* = 8.1 Hz, 1H), 7.03 (d, *J* = 8.7 Hz, 1H), 6.76 (d, *J* = 8.7 Hz, 1H), 4.72 (s, 2H), 3.82 (s, 3H), 3.73 (t, *J* = 6.1 Hz, 2H), 2.92–2.88 m, 6H), 2.65 (br, 4H), 2.39 (s, 3H), 2.25 (s, 3H). HRMS (ESI^+^): [M + H]^+^ calculated for C_28_H_33_ClN_5_O_2_ 506.2317, found: 506.2310.

##### 4.1.5.5 5-Methoxy-8-(4-methylpiperazin-1-yl)-N-(5-(4-(trifluoromethoxy)phenyl)pyridin-2-yl)-3,4-dihydroisoquinoline-2(1H)-carboxamide **(37)**

From **58** and **92** (R = 4-OCF_3_). 79% yield; mp 196–198 °C. HPLC 99.8%. ^1^H NMR (CDCl_3_) *δ* 8.41 (dd, *J* = 2.4, 0.5 Hz, 1H), 8.16 (dd, *J* = 8.7, 0.6 Hz, 1H), 7.84 (dd, *J* = 8.7, 2.5 Hz, 1H), 7.56 (AB br d, *J* = 8.8 Hz, 2H), 7.54 (s, 1H, NH), 7.30 (d, *J* = 8.0 Hz, 2H), 7.04 (d, *J* = 8.7 Hz, 1H), 6.75 (d, *J* = 8.7 Hz, 1H), 4.73 (s, 2H), 3.81 (s, 3H), 3.73 (t, *J* = 6.1 Hz, 2H), 2.91–2.88 m, 6H), 2.64 (br, 4H), 2.39 (s, 3H). HRMS (ESI^+^): [M + H]^+^ calculated for C_28_H_31_F_3_N_5_O_3_ 542.2374, found: 542.2368.

Method II

##### 4.1.5.6 5-Methoxy-8-(4-methylpiperazin-1-yl)-N-(5-(pyrimidin-5-yl)pyridin-2-yl)-3,4-dihydroisoquinoline-2(1H)-carboxamide **(31)**

Pyridine (1.2 mL, 6.6 mmol) was added to a solution of 2-amino-5-bromopyridine (**90**, 1.035 g, 5.982 mmol) in acetonitrile (20 mL) at 2 °C, followed by 4-nitrophenylchloroformate (1.326 g, 6.581 mmol). The resulting white slurry was stirred overnight. The white solids were filtered off, washed with DCM, dried under ambient conditions to give 4-nitrophenyl (5-bromopyridin-2-yl)carbamate (**93,** ~1.5 g); mp 236–239 °C. No mass spectroscopic and NMR data could be obtained for this compound due to the low solubility in suitable solvents.

A typical procedure is given for the synthesis of **94**: A mixture of **58** (0.13 g, 0.50 mmol) and **93** (0.254 g, 0.751 mmol) in DMF (3.5 mL) was stirred at 75 °C for 25 h. The mixture was diluted in water, then extracted with DCM (3x). The combined organic extract was washed with water, brine, dried (MgSO_4_) and concentrated to yield the crude product as a brown oil. The crude product was chromatographed using 0–15% MeOH in DCM to provide *N*-(5-bromopyridin-2-yl)-5-methoxy-8-(4-methylpiperazin-1-yl)-3,4-dihydroisoquinoline-2(1*H*)-carboxamide (**94**: X = OMe) as an off-white foam (166 mg, 72%); mp 76–79 °C. HPLC 92.8%. ^1^H NMR (CDCl_3_) *δ* 8.25 (dd, *J* = 2.4, 0.5 Hz, 1H), 8.02 (dd, *J* = 9.0, 0.6 Hz, 1H), 7.73 (dd, *J* = 9.0, 2.4 Hz, 1H), 7.28 (s, 1H), 7.03 (d, *J* = 8.7 Hz, 1H), 6.75 (d, *J* = 8.7 Hz, 1H), 4.69 (s, 2H), 3.81 (s, 3H), 3.69 (t, *J* = 6.1 Hz, 2H), 2.88 (apparent t, 6H), 2.63 (br, 4H), 2.38 (s, 3H). HRMS (ESI^+^): [M + H]^+^ calculated for C_21_H_27_BrN_5_O_2_ [^79^Br] 460.1343, found: 460.1341.

A mixture of (**94:** X = OMe) (0.138 mmol), pyrimidin-5-ylboronic acid (**73**: R = 3,5-diaza) (0.413 mmol) and 2 M Na_2_CO_3_ solution (0.590 mmol) in a mixture of toluene and EtOH (2:1, 3 mL) was purged with nitrogen. PdCl_2_dppf (5 mol%) was added, the mixture was purged again with nitrogen and heated at 85 °C overnight. The mixture was then diluted with water and extracted with EtOAc (3x). The combined organic extract was washed with brine, dried (MgSO_4_) and concentrated under reduced pressure to afford the crude product. This was chromatographed on silica using mixtures of MeOH and DCM to yield clean **31** (55%); mp 201–204 °C. HPLC 93.7%. ^1^H NMR (CDCl_3_) *δ* 9.22 (s, 1H), 8.94 (s, 2H), 8.45 (dd, *J* = 2.5, 0.7 Hz, 1H), 8.25 (dd, *J* = 8.7, 0.7 Hz, 1H), 7.88 (dd, *J* = 8.8, 2.5 Hz, 1H), 7.44 (s, 1H, NH), 7.05 (d, *J* = 8.7 Hz, 1H), 6.76 (d, *J* = 8.7 Hz, 1H), 4.73 (s, 2H), 3.82 (s, 3H), 3.74 (t, *J* = 6.1 Hz, 2H), 2.93–2.90 m, 6H), 2.65 (br, 4H), 2.40 (s, 3H). HRMS (ESI^+^): [M + H]^+^ calculated for C_25_H_30_N_7_O_2_ 460.2461, found: 460.2444.

##### 4.1.5.7 5-Methoxy-N-(5-(4-methoxyphenyl)pyridin-2-yl)-8-(4-methylpiperazin-1-yl)-3,4-dihydroisoquinoline-2(1H)-carboxamide **(33)**

From **94 (**X = OMe) and **73** (R = 4-OMe). 63% yield; mp 207–209 °C. HPLC 97.1%. ^1^H NMR (CDCl_3_) *δ* 8.39 (dd, *J* = 2.5, 0.6 Hz, 1H), 8.10 (dd, *J* = 8.7, 0.6 Hz, 1H), 7.82 (dd, *J* = 8.7, 2.5 Hz, 1H), 7.47 (AB br d, *J* = 8.8 Hz, 2H), 7.40 (s, 1H, NH), 7.03 (d, *J* = 8.7 Hz, 1H), 6.98 (AB br d, *J* = 8.8 Hz, 2H), 6.75 (d, *J* = 8.7 Hz, 1H), 4.72 (s, 2H), 3.85 (s, 3H), 3.81 (s, 3H), 3.72 (t, *J* = 6.1 Hz, 2H), 2.91–2.88 m, 6H), 2.64 (br, 4H), 2.39 (s, 3H). HRMS (ESI^+^): [M + H]^+^ calculated for C_28_H_34_N_5_O_3_ 488.2656, found: 488.2665.

##### 4.1.5.8 N-(5-(2-Chloro-4-(trifluoromethyl)phenyl)pyridin-2-yl)-5-methoxy-8-(4-methylpiperazin-1-yl)-3,4-dihydroisoquinoline-2(1H)-carboxamide **(34)**

From **94** (X = OMe) and **73** (X = 2-Cl, 4-CF_3_**)**. 15% yield; mp 61–64 °C. HPLC 92.6%. ^1^H NMR (CDCl_3_) *δ* 8.29 (dd, *J* = 2.4, 0.8 Hz, 1H), 8.19 (dd, *J* = 8.6, 0.6 Hz, 1H), 7.79–7.75 (m, 2H), 7.69 (s, 1H, NH), 7.58 (dd, *J* = 8.0, 0.8 Hz, 1H), 7.44 (d, *J* = 8.0 Hz, 1H), 7.03 (d, *J* = 8.8 Hz, 1H), 6.74 (d, *J* = 8.8 Hz, 1H), 4.73 (s, 2H), 3.80 (s, 3H), 3.73 (t, *J* = 6.0 Hz, 2H), 2.91–2.88 m, 6H), 2.64 (br, 4H), 2.39 (s, 3H). HRMS (ESI^+^): [M + H]^+^ calculated for C_28_H_30_ClF_3_N_5_O_2_ 560.2035, found: 560.2036.

##### 4.1.5.9 N-(5-(2,4-Dichlorophenyl)pyridin-2-yl)-5-methoxy-8-(4-methylpiperazin-1-yl)-3,4-dihydroisoquinoline-2(1H)-carboxamide **(36)**

From **94 (**X = OMe) and **73** (X = 2,4-diCl**)**. 55% yield; mp 108–111 °C. HPLC 91.8%. ^1^H NMR (CDCl_3_) *δ* 8.26 (dd, *J* = 2.4, 0.7 Hz, 1H), 8.14 (dd, *J* = 8.7, 0.7 Hz, 1H), 7.73 (dd, *J* = 8.7, 2.4 Hz, 1H), 7.50 (d, *J* = 2.0 Hz, 1H), 7.44 (s, 1H, NH), 7.32 (dd, *J* = 8.2, 2.0 Hz, 1H), 7.26 (d, *J* = 8.3 Hz, 1H), 7.04 (d, *J* = 8.7 Hz, 1H), 6.75 (d, *J* = 8.7 Hz, 1H), 4.72 (s, 2H), 3.81 (s, 3H), 3.72 (t, *J* = 6.1 Hz, 2H), 2.91–2.88 m, 6H), 2.65 (br, 4H), 2.40 (s, 3H). HRMS (ESI^+^): [M + H]^+^ calculated for C_27_H_30_Cl_2_N_5_O_2_ 526.1771, found: 526.1766.

##### 4.1.5.10 N-(5-(3,5-Bis(trifluoromethyl)phenyl)pyridin-2-yl)-5-methoxy-8-(4-methylpiperazin-1-yl)-3,4-dihydroisoquinoline-2(1H)-carboxamide **(38)**

From **94 (**X = OMe) and **73** (R = 3,5-di-CF_3_). 54% yield; mp 106–109 °C. HPLC 97.7%. ^1^H NMR (CDCl_3_) *δ* 8.47 (d, *J* = 1.9 Hz, 1H), 8.22 (dd, *J* = 8.8, 0.5 Hz, 1H), 7.97 (s, 2H), 7.90 (dd, *J* = 8.8, 2.5 Hz, 1H), 7.87 (s, 1H), 7.45 (s, 1H, NH), 7.02 (d, *J* = 8.7 Hz, 1H), 6.75 (d, *J* = 8.7 Hz, 1H), 4.72 (s, 2H), 3.82 (s, 3H), 3.73 (t, *J* = 6.1 Hz, 2H), 2.96–2.90 m, 6H), 2.76 (br, 4H), 2.47 (s, 3H). (ESI^+^): [M + H]^+^ calculated for C_29_H_30_F_6_N_5_O_2_ 594.2298, found: 594.2316.

The following compounds were similarly prepared:

##### 4.1.5.11 N-(5-(2,4-Dichlorophenyl)pyridin-2-yl)-5-methyl-8-(4-methylpiperazin-1-yl)-3,4-dihydroisoquinoline-2(1H)-carboxamide **(25)**

Following the typical procedure for **94** (X = OMe), *N*-(5-bromopyridin-2-yl)-5-methyl-8-(4-methylpiperazin-1-yl)-3,4-dihydroisoquinoline-2(1*H*)-carboxamide (**94**: X = Me) was prepared from **57** and **93**. It was purified by flash chromatography (2–15% MeOH in DCM) to give a yellow crystalline solid (71%); mp 117–120 °C. ^1^H NMR (CDCl_3_) *δ* 8.26 (dd, *J* = 2.5, 0.6 Hz, 1H), 8.05 (dd, *J* = 8.9, 0.6 Hz, 1H), 7.76 (dd, *J* = 9.0, 2.4 Hz, 1H), 7.28 (s, 1H, NH), 7.06 (d, *J* = 8.1 Hz, 1H), 6.93 (d, *J* = 8.4 Hz, 1H), 4.57 (s, 2H), 3.68 (t, *J* = 5.8 Hz, 2H), 2.95 (t, *J* = 5.3 Hz, 6H), 2.65 (br, 4H), 2.42 (s, 3H), 2.26 (s, 3H). MS (APCI): 444.1 [^79^Br], 446.1 [^81^Br] [M + H]^+^.

From **94** (X-Me) and **73** (R = 2,4-diCl) gave **25** (68% yield); mp 104–106 °C. HPLC 98.0%. ^1^H NMR (CDCl_3_) *δ* 8.27 (dd, *J* = 2.3, 0.7 Hz, 1H), 8.17 (dd, *J* = 8.7, 0.7 Hz, 1H), 7.76 (d, *J* = 8.7, 2.4 Hz, 1H), 7.51 (d, *J* = 2.0 Hz, 1H), 7.40 (s, 1H), 7.32 (dd, *J* = 8.3, 2.1 Hz, 1H), 7.28 (s, 1H, NH) 7.06 (d, *J* = 8.1 Hz, 1H), 6.93 (d, *J* = 8.1 Hz, 1H), 4.60 (s, 2H), 3.71 (t, *J* = 5.7 Hz, 2H), 2.97 (t, *J* = 5.7 Hz, 2H), 2.92 (t, *J* = 4.7 Hz, 4H), 2.59 (br, 4H), 2.38 (s, 3H), 2.26 (s, 3H). HRMS (ESI^+^): [M + H]^+^ calculated for C_27_H_30_Cl_2_N_5_O 510.18219, found: 510.18188.

##### 4.1.5.12 N-(5-(3,5-Bis(trifluoromethyl)phenyl)pyridin-2-yl)-5-methyl-8-(4-methylpiperazin-1-yl)-3,4-dihydroisoquinoline-2(1H)-carboxamide **(26)**

From **94** (X = Me) and **73** (R = 3,5-diCF_3_). 62% yield; mp 114–117 °C. HPLC 98.1%. ^1^H NMR (CDCl_3_) *δ* 8.48 (d, *J* = 1.9 Hz, 1H), 8.26 (dd, *J* = 7.9, 0.5 Hz, 1H), 7.98 (s, 2H), 7.92 (dd, *J* = 8.7, 2.5 Hz, 1H), 7.87 (s, 1H), 7.46 (s, 1H, NH), 7.06 (d, *J* = 8.1 Hz, 1H), 6.90 (d, *J* = 8.1 Hz, 1H), 4.61 (s, 2H), 3.72 (t, *J* = 5.8 Hz, 2H), 2.97 (t, *J* = 4.5 Hz, 6H), 2.72 (br, 4H), 2.46 (s, 3H), 2.28 (s, 3H). HRMS (ESI^+^): [M + H]^+^ calculated for C_29_H_30_F_6_N_5_O 578.23545, found: 578.2345.

##### 4.1.5.13 5-Ethyl-8-(4-methylpiperazin-1-yl)-N-(5-(4-(trifluoromethyl)phenyl)pyridin-2-yl)-3,4-dihydroisoquinoline-2(1H)-carboxamide **(27)**

Following the typical procedure for **94** (X = OMe), *N*-(5-bromopyridin-2-yl)-5-ethyl-8-(4-methylpiperazin-1-yl)-3,4-dihydroisoquinoline-2(1*H*)-carboxamide (**94**: X = Et) was prepared from **62** and **93**. It was purified by flash chromatography (0–10% MeOH in DCM) to give a yellow crystalline solid (70%); mp 73–76 °C. ^1^H NMR (CDCl_3_) *δ* 8.25 (d, *J* = 2.5 Hz, 1H), 8.01 (dd, *J* = 8.9, 0.4 Hz, 1H), 7.73 (dd, *J* = 9.0, 2.5 Hz, 1H), 7.29 (s, 1H, NH), 7.11 (d, *J* = 8.2 Hz, 1H), 7.01 (d, *J* = 8.2 Hz, 1H), 4.70 (s, 2H), 3.71 (t, *J* = 6.1 Hz, 2H), 2.92 (t, *J* = 4.7 Hz, 6H), 2.64–2.55 (m, 6H), 2.38 (s, 3H), 1.19 (t, *J* = 7.6 Hz, 3H). MS (APCI): 458.2 [^79^Br], 460.2 [^81^Br] [M + H]^+^.

Reaction of **94** (X = Et) and **73** (R = 4-CF_3_) as above gave **27** (35% yield); mp 190–192 °C. HPLC 97.2%. ^1^H NMR (CDCl_3_) *δ* 8.46 (dd, *J* = 2.4, 0.6 Hz, 1H), 8.19 (dd, *J* = 8.7, 0.6 Hz, 1H), 7.92 (dd, *J* = 8.7, 2.5 Hz, 1H), 7.71 (AB br d, *J* = 8.4 Hz, 2H), 6.66 (AB br d, *J* = 8.4 Hz, 2H), 7.40 (s, 1H, NH), 7.12 (d, *J* = 8.2 Hz, 1H), 7.02 (d, *J* = 8.2 Hz, 1H), 4.74 (s, 2H), 3.75 (t, *J* = 6.1 Hz, 2H), 2.93 (apparent t, 6H), 2.65–2.59 (m, 6H), 2.39 (s, 3H), 1.20 (t, *J* = 7.5 Hz, 3H). HRMS (ESI + ): [M + H] + calculated for C_29_H_33_F_3_N_5_O 524.2610, found: 524.2632.

##### 4.1.5.14 N-(5-(4-Chloro-2-methylphenyl)pyridin-2-yl)-5-ethyl-8-(4-methylpiperazin-1-yl)-3,4-dihydroisoquinoline-2(1H)-carboxamide **(28)**

From **94** (X = Et) and **81**. 71% yield; mp 180–182 °C. HPLC 94.0%. ^1^H NMR (CDCl_3_) *δ* 8.14 (dd, *J* = 2.4, 0.6 Hz, 1H), 8.12 (dd, *J* = 8.6, 0.6 Hz, 1H), 7.60 (dd, *J* = 8.6, 2.4 Hz, 1H), 7.37(s, 1H, NH), 7.28 (d, *J* = 2.1 Hz, 1H), 7.23 (dd, *J* = 8.1, 2.0 Hz, 1H), 7.14–7.11 (apparent t, 2H), 7.02 (d, *J* = 8.2 Hz, 1H), 4.74 (s, 2H), 3.74 (t, *J* = 6.1 Hz, 2H), 2.93 (apparent t, 6H), 2.65–2.59 (m, 6H), 2.39 (s, 3H), 2.25 (s, 3H), 1.19 (t, *J* = 7.5 Hz, 3H). HRMS (ESI^+^): [M + H]^+^ calculated for C_29_H_35_ClN_5_O 504.2525, found: 504.2534.

##### 4.1.5.15 5-Benzyl-N-(5-(4-chloro-2-methylphenyl)pyridin-2-yl)-8-(4-methylpiperazin-1-yl)-3,4-dihydroisoquinoline-2(1H)-carboxamide **(29)**

Following the typical procedure for **94** (X = OMe), 5-benzyl-*N*-(5-bromopyridin-2-yl)-8-(4-methylpiperazin-1-yl)-3,4-dihydroisoquinoline-2(1*H*)-carboxamide (**94**: X = Bn) was prepared from **63** and **93**. It was purified by flash chromatography (2–7% MeOH in DCM) to give a cream foamy solid (83%); mp 74–77 °C. ^1^H NMR (CDCl_3_) *δ* 8.24 (dd, *J* = 2.4, 0.6 Hz, 1H), 8.00 (dd, *J* = 8.9, 0.6 Hz, 1H), 7.73 (dd, *J* = 9.0, 2.4 Hz, 1H), 7.29–7.25 (m, 2H), 7.21–7.17 (m, 2H), 7.07–7.10 (m, 3H), 7.01 (d, *J* = 8.2 Hz, 1H), 4.69 (s, 2H), 3.97 (s, 2H), 3.59 (t, *J* = 6.1 Hz, 2H), 2.93 (t, *J* = 4.7 Hz, 4H), 2.81 (t, *J* = 6.1 Hz, 2H), 2.65 (br, 4H), 2.38 (s, 3H). MS (APCI): 520.2 [^79^Br], 522.1 [^81^Br] [M + H]^+^.

Reaction of **94** (X = Bn) and **81** as above gave **29** (49% yield); mp 107–110 °C. HPLC 96.5%. ^1^H NMR (CDCl_3_) *δ* 8.13 (dd, *J* = 2.3, 0.6 Hz, 1H), 8.09 (dd, *J* = 8.6, 0.6 Hz, 1H), 7.59 (dd, *J* = 8.6, 2.4 Hz, 1H), 7.29–7.27 (m, 3H), 7.24–7.18 (m, 2H), 7.14–7.07 (m, 4H), 7.02 (d, *J* = 8.2 Hz, 1H), 4.73 (s, 2H), 3.98 (s,2H), 3.63 (t, *J* = 6.1 Hz, 2H), 2.95 (t, *J* = 4.4 Hz, 4H), 2.83 (t, *J* = 6.1 Hz, 2H), 2.66 (br, 4H), 2.39 (s, 3H), 2.25 (s, 3H). HRMS (ESI^+^): [M + H]^+^ calculated for C_34_H_37_ClN_5_O 566.2681, found: 566.2663.

##### 4.1.5.16 5-Benzyl-N-(5-(2,4-dimethylphenyl)pyridin-2-yl)-8-(4-methylpiperazin-1-yl)-3,4-dihydroisoquinoline-2(1H)-carboxamide **(30)**

From **94** (X = Bn) and **73** (R = 2,4-diMe). 35% yield; mp 94–97 °C. HPLC 97.2%. ^1^H NMR (CDCl_3_) *δ* 8.16 (dd, *J* = 2.3, 0.6 Hz, 1H), 8.07 (dd, *J* = 8.6, 0.6 Hz, 1H), 7.62 (dd, *J* = 8.6, 2.4 Hz, 1H), 7.29–7.27 (m, 3H), 7.21–7.18 (m, 1H), 7.11–7.05 (m, 6H), 7.02 (d, *J* = 8.2 Hz, 1H), 4.73 (s, 2H), 3.98 (s,2H), 3.63 (t, *J* = 6.1 Hz, 2H), 2.95 (t, *J* = 4.4 Hz, 4H), 2.83 (t, *J* = 6.1 Hz, 2H), 2.66 (br, 4H), 2.39 (s, 3H), 2.36 (s, 3H), 2.24 (s, 3H). HRMS (ESI^+^): [M + H]^+^ calculated for C_35_H_40_N_5_O 546.3227, found: 546.3214.

The following compounds were similarly prepared:

##### 4.1.5.17 N-(5-(3,5-Bis(trifluoromethyl)phenyl)pyridin-2-yl)-8-(4-methylpiperazin-1-yl)-5-(methylthio)-3,4-dihydroisoquinoline-2(1H)-carboxamide **(39)**

Following the typical procedure for **94** (X = OMe), *N*-(5-bromopyridin-2-yl)-8-(4-methylpiperazin-1-yl)-5-(methylthio)-3,4-dihydroisoquinoline-2(1*H*)-carboxamide (**94**: X = SMe) was prepared from **61** and **93**. It was purified by flash chromatography (0–15% MeOH in DCM) to give an off-white solid (71%); mp 160–162 °C. ^1^H NMR (CDCl_3_) *δ* 8.25 (dd, *J* = 2.4, 0.5 Hz, 1H), 8.00 (dd, *J* = 8.9, 0.6 Hz, 1H), 7.74 (dd, *J* = 9.0, 2.4 Hz, 1H), 7.29 (s, 1H, NH), 7.18 (d, *J* = 8.4 Hz, 1H), 7.05 (d, *J* = 8.4 Hz, 1H), 4.69 (s, 2H), 3.71 (t, *J* = 6.2 Hz, 2H), 3.00 (t, *J* = 6.1 Hz, 2H), 2.94 (t, *J* = 4.6 Hz, 4H), 2.67 (br, 4H), 2.43 (s, 3H), 2.40 (s, 3H). MS (APCI): 476.1 [^79^Br], 478.1 [^81^Br], [M + H]+.

Reaction of **94** (X = SMe) and **73** (R = 3,5-diOCF_3_) gave **39** (58% yield); mp 106–109 °C. HPLC 94.5%. ^1^H NMR (CDCl_3_) *δ* 8.47 (dd, *J* = 2.5, 0.6 Hz, 1H), 8.22 (dd, *J* = 8.8, 0.6 Hz, 1H), 7.97 (s, 2H), 7.90 (dd, *J* = 8.8, 2.5 Hz, 1H), 7.87 (s, 1H), 7.45 (s, 1H, NH), 7.17 (d, *J* = 8.2 Hz, 1H), 7.04 (d, *J* = 8.4 Hz, 1H), 4.72 (s, 2H), 3.76 (t, *J* = 6.2 Hz, 2H), 3.03 (t, *J* = 6.2 Hz, 2H), 2.98 (t, *J* = 4.6 Hz, 4H), 2.76 (br, 4H), 2.46 (s, 3H), 2.44 (s, 3H). HRMS (ESI^+^): [M + H]^+^ calculated for C_29_H_30_F_6_N_5_OS 610.2075, found: 610.2062.

##### 4.1.5.18 N-(5-(4-Chloro-2-methylphenyl)pyridin-2-yl)-8-(4-methylpiperazin-1-yl)-5-(methylthio)-3,4-dihydroisoquinoline-2(1H)-carboxamide **(40)**

From **94** (X = SMe) and **81**. 54% yield. mp 111–114 °C. HPLC 95.2%. ^1^H NMR (CDCl_3_) *δ* 8.15 (dd, *J* = 2.4, 0.7 Hz, 1H), 8.11 (dd, *J* = 8.6, 0.7 Hz, 1H), 7.60 (dd, *J* = 8.6, 2.4 Hz, 1H), 7.34 (s, 1H, NH), 7.28 (d, *J* = 2.1 Hz, 1H), 7.24–7.18 (m, 2H), 7.13 (d, *J* = 8.2 Hz, 1H), 7.06 (d, *J* = 8.4 Hz, 1H), 4.73 (s, 2H), 3.75 (t, *J* = 6.1 Hz, 2H), 3.02 (t, *J* = 6.1 Hz, 2H), 2.93 (t, *J* = 4.6 Hz, 4H), 2.65 (br, 4H), 2.44 (s, 3H), 2.39 (s, 3H), 2.25 (s, 3H). HRMS (ESI^+^): [M + H]^+^ calculated for C_28_H_33_ClN_5_OS 522.2094, found: 522.2077.

##### 4.1.5.19 N-(5-(4-Chloro-3-(trifluoromethyl)phenyl)pyridin-2-yl)-5-fluoro-8-(4-methylpiperazin-1-yl)-3,4-dihydroisoquinoline-2(1H)-carboxamide **(41)**

Following the typical procedure for **94** (X = OMe), *N*-(5-bromopyridin-2-yl)-5-fluoro-8-(4-methylpiperazin-1-yl)-3,4-dihydroisoquinoline-2(1*H*)-carboxamide (**94**: X = F) was prepared from **59** and **93**. It was purified by flash chromatography (2–15% MeOH in DCM) to give a white foamy solid (71%); mp 160–162 °C. ^1^H NMR (CDCl_3_) *δ* 8.26 (dd, *J* = 2.4, 0.6 Hz, 1H), 8.01 (dd, *J* = 8.9, 0.6 Hz, 1H), 7.75 (dd, *J* = 9.0, 2.4 Hz, 1H), 7.30 (s, 1H, NH), 7.01 (dd, *J* = 8.7, 4.9 Hz, 1H), 6.94 (t, *J* = 8.8 Hz, 1H), 4.69 (s, 2H), 3.72 (t, *J* = 6.1 Hz, 2H), 2.94 (t, *J* = 6.1 Hz, 2H), 2.90 (t, *J* = 4.7 Hz, 4H), 2.63 (br, 4H), 2.38 (s, 3H). MS (APCI): 448.3 [^79^Br], 450.3 [^81^Br] [M + H]^+^.

Reaction of **94** (X = F) and **73** (R = 3-CF_3_, 4-Cl) gave **41** (30% yield); mp 97–100 °C. HPLC 93.5%. ^1^H NMR (CDCl_3_) *δ* 8.43 (dd, *J* = 2.5, 0.6 Hz, 1H), 8.18 (dd, *J* = 8.8, 0.6 Hz, 1H), 7.87–7.84 (m, 2H), 7.65 (dd, *J* = 8.3, 2.1 Hz, 1H), 7.59 (d, *J* = 8.3 Hz, 1H), 7.42 (s, 1H, NH), 7.04–7.00 (m, 1H), 6.95 (t, *J* = 8.7 Hz, 1H), 4.73 (s, 2H), 3.76 (t, *J* = 6.1 Hz, 2H), 2.96 (t, *J* = 6.1 Hz, 2H), 2.92 (t, *J* = 4.6 Hz, 4H), 2.66 (br, 4H), 2.40 (s, 3H). HRMS (ESI^+^): [M + H]^+^ calculated for C_27_H_27_ClF_4_N_5_O 548.18348, found: 548.18230.

##### 4.1.5.20 N-(5-(4-Chloro-2-methylphenyl)pyridin-2-yl)-5-fluoro-8-(4-methylpiperazin-1-yl)-3,4-dihydroisoquinoline-2(1H)-carboxamide **(42**)

From **94** (X = F) and **81**. 57% yield; mp 98–101 °C. HPLC 93.2%. ^1^H NMR (CDCl_3_) *δ* 8.15 (dd, *J* = 2.4, 0.7 Hz, 1H), 8.10 (dd, *J* = 8.6, 0.7 Hz, 1H), 7.61 (dd, *J* = 8.6, 2.3 Hz, 1H), 7.34 (s, 1H, NH), 7.28 (d, *J* = 2.1 Hz, 1H), 7.24 (dd, *J* = 8.4, 2.0 Hz, 1H), 7.14 (d, *J* = 8.2 Hz, 1H), 7.04–7.00 (m, 1H), 6.94 (t, *J* = 8.7 Hz, 1H), 4.73 (s, 2H), 3.76 (t, *J* = 6.1 Hz, 2H), 2.95 (t, *J* = 6.0 Hz, 2H), 2.91 (t, *J* = 4.4 Hz, 4H), 2.64 (br, 4H), 2.39 (s, 3H), 2.26 (s, 3H). HRMS (ESI^+^): [M + H]^+^ calculated for C_27_H_30_ClFN_5_O 494.21174, found: 494.21231.

#### 4.1.6 Preparation of the –COCH_2_-linked pyridyl side compounds **43**–**47** of Scheme 5 and Table 1

A mixture of (4-methylpiperazin-1-yl)isoquinoline (**56**) (119.0 mg, 0.514 mmol), 2-(5-bromopyridin-2-yl)acetic acid (**96**) (111.1 mg, 0.514 mmol), dimethylamino-4-pyridine (62.8 mg, 0.514 mmol), *N*-hydroxybenzotriazole (69.5 mg, 0.514 mmol) and diisopropylethylamine (0.30 mL, 1.80 mmol) in anhydrous *N*,*N*-dimethylformamide (7 mL) was treated under nitrogen at room temperature with EDCI hydrochloride (167.5 mg, 0.874 mmol). The mixture was stirred at room temperature overnight, it was then diluted with water. The aqueous mixture was extracted with DCM (3x) and the combined extract was washed with water, brine, dried (MgSO_4_) and concentrated under reduced pressure. The crude product was chromatographed on silica using 3–9% MeOH in DCM to afford 2-(5-bromopyridin-2-yl)-1-(8-(4-methylpiperazin-1-yl)-3,4-dihydroisoquinolin-2(1*H*)-yl)ethan-1-one (**97**) as a yellow oil (0.188 g, 85%). ^1^H NMR (CDCl_3_) δ (1:1 mixture of rotamers, two sets of data). 8.59 (d, *J* = 2.0 Hz, 1H), 8.56 (d, *J* = 2.1 Hz, 1H), 7.77 (dd, *J* = 8.3, 2.4 Hz, 1H), 7.72 (dd, *J* = 8.3, 2.4 Hz, 1H), 7.30 (d, *J* = 8.4 Hz, 1H), 7.23–7.16 (m, 3H), 6.97 (t, *J* = 7.8 Hz, 2H), 6.92–6.87 (m, 2H), 4.75 (s, 2H), 4.66 (s, 2H), 4.00 (s, 2H), 3.94 (s, 2H), 3.81–3.76 (m, 4H), 2.92 (t, *J* = 4.7 Hz, 4H), 2.88–2.82 (m, 8H), 2.61 (br, 8H), 2.38 (s, 3H), 2.35 (s, 3H). MS (APCI): 429.1 [^79^Br], 431.2 [^81^Br], [M + H]^+^.

Reaction of **97** with the appropriate boronic acids (**73**) or **81** using the reaction conditions and purification procedures described above for **31**, gave the following compounds of Table 1:

##### 4.1.6.1 2-(5-(4-Chlorophenyl)pyridin-2-yl)-1-(8-(4-methylpiperazin-1-yl)-3,4-dihydroisoquinolin-2(1H)-yl)ethan-1-one **(43)**

M.p. 49–52 °C. HPLC 93.4%. ^1^H NMR (CDCl_3_) δ (1:1 mixture of rotamers, two sets of data). 8.72 (d, *J* = 2.0 Hz, 1H), 8.70 (d, *J* = 2.1 Hz, 1H), 7.81 (dd, *J* = 8.1, 2.4 Hz, 1H), 7.76 (dd, *J* = 8.1, 2.4 Hz, 1H), 7.52–7.42 (m, 9H), 7.37 (d, *J* = 8.1 Hz, 1H), 7.21–7.15 (m, 2H), 7.01–6.96 (m, 2H), 6.93–6.87 (m, 2H), 4.77 (s, 2H), 4.71 (s, 2H), 4.10 (s, 2H), 4.03 (s, 2H), 3.85–3.81 (m, 4H), 2.93–2.84 (m, 12H), 2.60 (br, 8H), 2.36 (s, 3H), 2.35 (s, 3H). HRMS (ESI^+^): [M + H]^+^ calculated for C_27_H_30_ClN_4_O 461.2103, found: 461.2101.

##### 4.1.6.2 1-(8-(4-Methylpiperazin-1-yl)-3,4-dihydroisoquinolin-2(1H)-yl)-2-(5-phenylpyridin-2-yl)ethan-1-one **(44)**

Semi-solid. HPLC 94.4%. ^1^H NMR (CDCl_3_) δ (1:1 mixture of rotamers, two sets of data). 8.76 (d, *J* = 2.0 Hz, 1H), 8.74 (d, *J* = 2.1 Hz, 1H), 7.85 (dd, *J* = 8.1, 2.4 Hz, 1H), 7.80 (dd, *J* = 8.1, 2.4 Hz, 1H), 7.58–7.52 (m, 4H), 7.49–7.44 (m, 5H), 7.42–7.35 (m, 3H), 7.19–7.15 (m, 2H), 7.01–6.96 (m, 2H), 6.93–6.87 (m, 2H), 4.78 (s, 2H), 4.71 (s, 2H), 4.10 (s, 2H), 4.04 (s, 2H), 3.85–3.81 (m, 4H), 2.93–2.90 (m, 6H), 2.90–2.83 (m, 6H), 2.60 (br, 8H), 2.36 (s, 3H), 2.34 (s, 3H). HRMS (ESI^+^): [M + H]^+^ calculated for C_27_H_31_N_4_O 427.2492, found: 427.2486.

##### 4.1.6.3 2-(5-(3-Chlorophenyl)pyridin-2-yl)-1-(8-(4-methylpiperazin-1-yl)-3,4-dihydroisoquinolin-2(1H)-yl)ethan-1-one **(45)**

Semi-solid. HPLC 94.7%. ^1^H NMR (CDCl_3_) δ (1:1 mixture of rotamers, two sets of data). 8.73 (d, *J* = 2.0 Hz, 1H), 8.70 (d, *J* = 2.1 Hz, 1H), 7.82 (dd, *J* = 8.1, 2.4 Hz, 1H), 7.77 (dd, *J* = 8.1, 2.4 Hz, 1H), 7.57–7.37 (m, 10*H*), 7.21–7.15 (m, 2H), 7.01–6.96 (m, 2H), 6.93–6.87 (m, 2H), 4.77 (s, 2H), 4.71 (s, 2H), 4.11 (s, 2H), 4.04 (s, 2H), 3.85–3.81 (m, 4H), 2.94–2.84 (m, 12H), 2.61 (br, 8H), 2.37 (s, 3H), 2.35 (s, 3H). HRMS (ESI^+^): [M + H]^+^ calculated for C_27_H_30_ClN_4_O 461.2103, found: 461.2095.

##### 4.1.6.4 1-(8-(4-Methylpiperazin-1-yl)-3,4-dihydroisoquinolin-2(1H)-yl)-2-(5-(4-(trifluoromethoxy)phenyl)pyridin-2-yl)ethan-1-one **(46)**

Semi-solid. HPLC 95.2%. ^1^H NMR (CDCl_3_) δ (1:1 mixture of rotamers, two sets of data). 8.73 (d, *J* = 2.2 Hz, 1H), 8.70 (d, *J* = 2.2 Hz, 1H), 7.82 (dd, *J* = 8.1, 2.4 Hz, 1H), 7.77 (dd, *J* = 8.1, 2.4 Hz, 1H), 7.59–7.52 (m, 4H), 7.37 (d, *J* = 8.1 Hz, 1H), 7.39 (d, *J* = 8.1 Hz, 1H), 7.32 (d, *J* = 8.5 Hz, 4H), 7.21–7.15 (m, 2H), 7.01–6.96 (m, 2H), 6.93–6.87 (m, 2H), 4.78 (s, 2H), 4.72 (s, 2H), 4.10 (s, 2H), 4.04 (s, 2H), 3.85–3.81 (m, 4H), 2.93–2.84 (m, 12H), 2.60 (br, 8H), 2.36 (s, 3H), 2.34 (s, 3H). HRMS (ESI^+^): [M + H]^+^ calculated for C_28_H_30_F_3_N_4_O_2_ 511.2315, found: 511.2312.

##### 4.1.6.5 (2-(5-(4-Chloro-2-methylphenyl)pyridin-2-yl)-1-(8-(4-methylpiperazin-1-yl)-3,4-dihydroisoquinolin-2(1H)-yl)ethan-1-one **(47)**

M.p. 45–48 °C. HPLC 95.9%. ^1^H NMR (CDCl_3_) δ (1:1 mixture of rotamers, two sets of data). 8.47 (d, *J* = 2.2 Hz, 1H), 8.45 (d, *J* = 2.2 Hz, 1H), 7.60–7.53 (m, 2H), 7.44 (d, *J* = 8.0 Hz, 1H), 7.37 (d, *J* = 8.0 Hz, 1H), 7.30–7.10 (m, 8H), 7.01–6.96 (m, 2H), 6.93–6.87 (m, 2H), 4.79 (s, 2H), 4.74 (s, 2H), 4.10 (s, 2H), 4.04 (s, 2H), 3.86–3.83 (m, 4H), 2.94–2.84 (m, 12H), 2.60 (br, 8H), 2.36 (s, 3H), 2.35 (s, 3H), 2.25 (s, 3H), 2.22 (s, 3H). HRMS (ESI^+^): [M+H]^+^ calculated for C_28_H_32_ClN_4_O 475.2259, found: 475.2244.

#### 4.1.7 Preparation of the carboxypiperidine-linked compounds **48**–**51** of Scheme 6 and Table 1

##### 4.1.7.1 (5-Methyl-8-(4-methylpiperazin-1-yl)-3,4-dihydroisoquinolin-2(1H)-yl)(4-(4-(trifluoromethoxy)phenoxy)piperidin-1-yl)methanone **(48)**

Et_3_N (0.837 mL, 6.00 mmol) was added to a suspension of 4-(4-(trifluoromethoxy)phenoxy)piperidine hydrochloride (**99**-HCl) (523 mg, 2.00 mmol) in dry DCM (7.5 mL) at 0 °C, followed by the addition of 4-nitrophenyl carbonochloridate (**98**) (484 mg, 2.40 mmol). The resulting mixture was allowed to warm to room temperature and was stirred for 3 h. The solvent was evaporated and the residue was dissolved in EtOAc, washed with 5% ammonia five times, then brine and dried over anhydrous Na_2_SO_4_. Removal of solvent gave 4-nitrophenyl 4-(4-(trifluoromethoxy)phenoxy)piperidine-1-carboxylate **(1 0 1**) as an off-white solid (772 mg, 90%). ^1^H NMR (CDCl_3_) *δ* 8.28–8.24 (m, 2H), 7.33–7.29 (m, 2H), 7.16 (d, *J* = 8.5 Hz, 2H), 6.95–6.91 (m, 2H), 4.59–4.54 (m, 1H), 3.88–3.62 (m, 4H), 3.38–3.31 (m, 2H), 1.98–1.88 (m, 2H); HRMS calcd. for C_19_H_17_F_3_N_2_O_6_ (M + H^+^) *m*/*z* 427.1113, found 427.1116.

A mixture of **57** (80 mg, 0.33 mmol), **101** (200 mg, 0.47 mmol), DIPEA (0.115 mL, 0.66 mmol) and DMAP (4 mg, 0.033 mmol) in toluene (5 mL) was heated in an oil bath at 110 °C for 3 days. The solvent was evaporated and the residue was taken in EtOAc and washed with 5% ammonia five times (until the aqueous phase became colourless). The organic phase was washed with brine, dried over anhydrous Na_2_SO_4_ and filtered through a pad of alumina (Aluminium Oxide 90, Standardised, Merck). The solvent was removed to give the crude product, which was purified by column chromatography (alumina), using gradient mixtures of EtOAc and hexanes (1:2, then 2:3) as the eluent, to give **48** as a white solid (53 mg, 30%). A sample was further purified by preparative HPLC (Column: Synergi-Max RP 4, 250 × 21.20 mm; Mobile phase: A/B = from 90% to 2% (A: 0.1% THA in water pH 2.56, B: 90% acetonitrile in water); flow rate 9 mL/ min, gradient method; wave length: 254 nm, 288 nm). HPLC 90.4%. M.p. 91–94 °C. ^1^H NMR (CDCl_3_) *δ* 7.14 (d, *J* = 8.4 Hz, 2H), 7.02 (d, *J* = 8.1 Hz, 1H), 6.93–6.89 (m, 3H), 4.50–4.45 (m, 1H), 4.35 (s, 2H), 3.60–3.54 (m, 2H), 3.46 (t, *J* = 5.6 Hz, 2H), 3.25–3.19 (m, 2H), 2.92–2.89 (m, 6H), 2.56 (br, 4H), 2.35 (s, 3H), 2.21 (s, 3H), 2.04–1.98 (m, 2H), 1.87–1.82 (m, 2H); HRMS calcd. for C_28_H_35_F_3_N_4_O_3_ (M + H^+^) *m*/*z* 533.2733, found 533.2743.

##### 4.1.7.2 (5-Methyl-8-(4-methylpiperazin-1-yl)-3,4-dihydroisoquinolin-2(1H)-yl)(4-(2-(trifluoromethyl)phenoxy)piperidin-1-yl)methanone **(49)**

A solution of 4-(2-(trifluoromethyl)phenoxy)piperidine (**100**) (500 mg, 2.04 mmol) in dry DCM (5 mL) was cooled in an ice bath, then treated with triethylamine (0.500 mL, 3.57 mmol), followed by **98** (493 mg, 2.45 mmol). The resulting mixture was allowed to warm up to room temperature and was stirred for 3 h. The solvent was evaporated and the residue was taken in EtOAc, and washed with 5% ammonia five times (until the aqueous phase became colourless). The solvent was removed to give the crude product, which was purified by column chromatography (silica), using a mixture of EtOAc and petrol hexanes (1:1) as the eluent, to give 4-nitrophenyl 4-(2-(trifluoromethyl)phenoxy)piperidine-1-carboxylate (**102**) as a colourless gum (618 mg, 74%). ^1^H NMR (CDCl_3_) *δ* 8.28–8.21 (m, 2H), 7.62–7.60 (m, 1H), 7.52–7.48 (m, 1H), 7.33–7.30 (m, 2H), 7.05–7.00 (m, 2H), 4.82–4.78 (m, 1H), 3.93–3.82 (m, 2H), 3.75–3.69 (m, 1H), 3.62–3.55 (m, 1H), 2.08–1.96 (m, 4H); HRMS calcd. for C_19_H_17_F_3_N_2_O_5_ (M + H^+^) *m*/*z* 411.1160, found 411.1168.

A mixture of **57** (80 mg, 0.33 mmol), **102** (200 mg, 0.49 mmol) and DIPEA (0.115 mL, 0.66 mmol) in toluene (5 mL) was heated in an oil bath at 100 °C for 48 h. The solvent was evaporated and the residue was taken in EtOAc and washed with 5% ammonia five times (until the aqueous phase became colourless). The organic phase was washed with brine, dried over anhydrous Na_2_SO_4_ and filtered through a pad of alumina. The solvent was removed to give the crude product, which was purified by column chromatography (alumina), using gradient mixtures of EtOAc and hexanes (1:1, then 2:1) as the eluent, followed by recrystallisation from ether and heptane to give **49** as a white solid (90 mg, 54%); mp 55–58 °C. HPLC 94.6%. ^1^H NMR (CDCl_3_) *δ* 7.58 (dd, *J* = 8.0, 1.1 Hz, 1H), 7.49–7.45 (m, 1H), 7.03–6.98 (m, 3H), 6.90 (d, *J* = 8.0 Hz, 1H), 4.72–4.68 (m, 1H), 4.34 (s, 2H), 3.55–3.50 (m, 2H), 3.45 (t, *J* = 5.6 Hz, 2H), 3.39–3.34 (m, 2H), 2.92–2.89 (m, 6H), 2.56 (br, 4H), 2.35 (s, 3H), 2.21 (s, 3H), 2.04–1.90 (m, 4H); HRMS calcd. for C_28_H_35_F_3_N_4_O_2_ (M + H^+^) *m*/*z* 517.2784, found 517.2795.

##### 4.1.7.3 (5-Methoxy-8-(4-methylpiperazin-1-yl)-3,4-dihydroisoquinolin-2(1H)-yl)(4-(4-(trifluoromethoxy)phenoxy)piperidin-1-yl)methanone **(50)**

A mixture of **58** (80 mg, 0.33 mmol), **101** (200 mg, 0.47 mmol), DIPEA (0.115 mL, 0.66 mmol) and DMAP (4 mg, 0.033 mmol) in toluene (5 mL) was heated in an oil bath at 110 °C for 3 days. Workup as above gave the crude product, which was purified by column chromatography packed with alumina, using gradient mixtures of EtOAc and petrol ether (v/v = 1:2, then 1:1) as eluents, followed by recrystallisation from ether and heptane to give **50** as a white solid (76 mg, 43%); mp 49–51 °C. HPLC 90.3%. ^1^H NMR (CDCl_3_) *δ* 7.14 (dd, *J* = 9.0, 0.7 Hz, 2H), 7.00 (d, *J* = 8.6 Hz, 1H), 6.92–6.88 (m, 2H), 6.71 (d, *J* = 8.7 Hz, 1H), 4.48–4.44 (m, 3H), 3.80 (s, 3H), 3.60–3.53 (m, 2H), 3.49 (t, *J* = 6.0 Hz, 2H), 3.48–3.17 (m, 2H), 2.92–2.89 (m, 4H), 2.81 (t, *J* = 6.0 Hz, 2H), 2.61 (br, 4H), 2.38 (s, 3H), 2.05–1.97 (m, 2H), 1.86–1.78 (m, 2H); HRMS calcd. for C_28_H_35_F_3_N_4_O_4_ (M + H^+^) *m*/*z* 549.2694, found 549.2697

##### 4.1.7.4 (5-Methoxy-8-(4-methylpiperazin-1-yl)-3,4-dihydroisoquinolin-2(1H)-yl)(4-(2-(trifluoromethyl)phenoxy)piperidin-1-yl)methanone **(51)**

A mixture of **58** (85 mg, 0.32 mmol), **102** (200 mg, 0.49 mmol) and Et_3_N (0.090 mL, 0.64 mmol) in MeCN (5 mL) of was heated in an oil bath at 70 °C for 48 h. Workup and purification as above gave **51** as a white solid (98 mg, 57%); mp 74–77 °C. HPLC 97.0%. ^1^H NMR (CDCl_3_) *δ* 7.58 (d, *J* = 7.0 Hz, 1H), 7.49–7.45 (m, 1H), 7.01–6.98 (m, 3H), 6.71 (d, *J* = 8.7 Hz, 1H), 4.68–4.66 (m, 1H), 4.45 (s, 2H), 3.80 (s, 3H), 3.55–3.48 (m, 4H), 3.34–3.29 (m, 2H), 2.87 (t, *J* = 4.6 Hz, 4H), 2.81 (t, *J* = 6.0 Hz, 2H), 2.58 (br, 4H), 2.36 (s, 3H), 2.03–1.97 (m, 2H), 1.94–1.87 (m, 2H); HRMS calcd. for C_28_H_35_F_3_N_4_O_3_ (M + H^+^) *m*/*z* 533.2733, found 533.2740.

#### 4.1.8 Preparation of the carbamate-linked compounds **52**–**55** of Scheme 7 and Table 2

##### 4.1.8.1 2-((5-(4-Chloro-2-methylphenyl)pyridin-2-yl)methyl)-5-methyl-8-(piperazin-1-yl)-1,2,3,4-tetrahydroisoquinoline **(52)**

A solution of **86** (0.230 g, 0.521 mmol) in PhMe (5 mL) was purged with nitrogen for 1 min. BINAP (0.017 g, 0.026 mmol), Pd_2_(dba)_3_ (0.007 g, 0.0076 mmol) and *N*-Bocpiperazine (0.146 g, 0.782 mmol) were then added, and the mixture stirred at 80 °C for 15 min. A bright orange solution formed, upon which NaO*^t^*Bu (0.075 g, 0.782 mmol) was added, and the mixture stirred at reflux under nitrogen for 18 h. The reaction was cooled and partitioned between EtOAc and water, and the organic phase was dried, filtered and evaporated. Column chromatography (1:1, x4:EtOAc) afforded *tert*-butyl 4-(2-((5-(4-chloro-2-methylphenyl)pyridin-2-yl)methyl)-5-methyl-1,2,3,4-tetrahydroisoquinolin-8-yl)piperazine-1-carboxylate **103** (0.099 g, 35%) as a yellow solid; mp 76–79 °C. ^1^H NMR (CDCl_3_) *δ* 8.52 (d, *J* = 1.5 Hz, 1H), 7.52–7.63 (m, 2H), 7.22–7.33 (m, 2H), 7.15 (d, *J* = 7.8 Hz, 1H), 7.00 (d, *J* = 8.0 Hz, 1H), 6.81 (d, *J* = 8.0 Hz, 1H), 3.91 (s, 2H), 3.78 (s, 2H), 3.43 (s, 4H), 2.88 (t, *J* = 5.4 Hz, 2H), 2.75–2.83 (m, 6H), 2.25 (s, 3H), 2.19 (s, 3H), 1.48 (s, 9H); Anal. (C_32_H_39_ClN_4_O_2_) C, H, N.

Trifluoroacetic acid (0.5 mL) was added to a solution of **103** (0.070 g, 0.128 mmol) in DCM (2.5 mL) at room temperature and the mixture stirred at this temperature for 2 h. Sat. NaHCO_3_ was added and the reaction was extracted with DCM. The organic phase was dried, filtered and evaporated to afford **52** as a yellow oil (0.025 g, 35%). ^1^H NMR (CDCl_3_) δ (tri-HCl salt) 8.69 (dd, *J* = 2.2 Hz, *J* = 0.8 Hz, 1H), 7.94 (dd, *J* = 8.0 Hz, *J* = 2.2 Hz, 1H), 7.67 (d, *J* = 8.0 Hz, 1H), 7.40 (d, *J* = 2.1 Hz, 1H), 7.32 (dd, *J* = 8.1 Hz, , *J* = 2.2 Hz, 1H), 7.23–7.39 (m, 2H), 7.14 (d, *J* = 8.1 Hz, 1H), 4.69 (s, 4H), 3.71 (s, 2H), 3.39 (s, 4H), 3.09–3.16 (m, 6H), 2.28 (s, 6H); Anal. (C_27_H_34_Cl_4_N_4_) C, H, N (tri-HCl salt).

##### 4.1.8.2 4-(2-((5-(4-Chloro-2-methylphenyl)pyridin-2-yl)methyl)-5-methyl-1,2,3,4-tetrahydroisoquinolin-8-yl)morpholine **(53)**

A solution of **86** (0.093 g, 0.21 mmol) in toluene (2 mL) was purged with nitrogen for 1 min. BINAP (0.007 g, 0.010 mmol), Pd_2_(dba)_3_ (0.005 g, 0.0053 mmol) and morpholine (0.027 mL, 0.316 mmol) were then added, and the mixture stirred at 80 °C for 15 min. A bright orange solution formed, upon which NaO*^t^*Bu (0.030 g, 0.316 mmol) was added, and the mixture stirred at reflux under nitrogen for 4 h. The reaction was cooled and partitioned between EtOAc and water, and the organic phase was dried, filtered and evaporated. Column chromatography (2:1, x4:EtOAc) afforded **53** (0.024 g, 26%) as a yellow oil, which was further purified by the general salt formation/back extraction procedure as described above. ^1^H NMR (CDCl_3_) *δ* 8.52 (dd, *J* = 2.0 Hz, *J* = 1.0 Hz, 1H), 7.58–7.62 (m, 2H), 7.22–7.32 (m, 2H), 7.15 (d, *J* = 7.8 Hz, 1H), 7.02 (d, *J* = 8.0 Hz, 1H), 6.87 (d, *J* = 8.0 Hz, 1H), 3.91 (s, 2H), 3.74–3.76 (m, 6H), 2.78–2.89 (m, 8H), 2.29 (s, 3H), 2.19 (s, 3H); Anal. (C_27_H_33_Cl_4_N_3_O) C, H, N (tri-HCl salt).

##### 4.1.8.3 4-(2-((5-(4-Chloro-2-methylphenyl)pyridin-2-yl)methyl)-5-methyl-1,2,3,4-tetrahydroisoquinolin-8-yl)yhiomorpholine **(54)**

A solution of **86** (0.071 g, 0.161 mmol) in toluene (2.5 mL) was purged with nitrogen for 1 min, treated with BINAP (0.005 g, 0.008 mmol), Pd_2_(dba)_3_ (0.004 g, 0.004 mmol) and thiomorpholine (0.022 mL, 0.241 mmol) as above, to give **54** as a yellow solid (0.053 g, 71%); mp 133–135 °C. ^1^H NMR (CDCl_3_) *δ* 8.53 (dd, *J* = 2.0 Hz, *J* = 0.8 Hz, 1H), 7.55–7.63 (m, 2H), 7.25–7.30 (m, 2H), 7.16 (d, *J* = 7.8 Hz, 1H), 7.01 (d, *J* = 8.0 Hz, 1H), 6.82 (d, *J* = 8.0 Hz, 1H), 3.90 (s, 2H), 3.72 (s, 2H), 3.06 (s, 4H), 2.85 (t, *J* = 5.5 Hz, 2H), 2.79 (t, *J* = 5.5 Hz, 2H), 2.27 (s, 3H), 2.15 (s, 3H); Anal. (C_27_H_30_ClN_3_S) C, H, N.

##### 4.1.8.4 2-((5-(4-Chloro-2-methylphenyl)pyridin-2-yl)methyl)-5-methyl-8-(piperidin-1-yl)-1,2,3,4-tetrahydroisoquinoline **(55)**

A solution of **104** (0.267 g, 1.18 mmol) in toluene (7.5 mL) was purged with nitrogen for 1 min. BINAP (0.037 g, 0.059 mmol), Pd_2_(dba)_3_ (0.054 g, 0.059 mmol) and piperidine (0.175 mL, 1.77 mmol) were then added, and the mixture stirred at 80 °C for 15 min. A bright orange solution formed, upon which NaO*^t^*Bu (0.170 g, 1.77 mmol) was added, and the mixture stirred at reflux under nitrogen for 4 h. The reaction was cooled and partitioned between EtOAc and water, and the organic phase was dried, filtered and evaporated. Column chromatography (4:1, x4:EtOAc) afforded 5-methyl-8-(piperidin-1-yl)isoquinoline (**105**) as a brown oil (0.169 g, 62%), which was used directly. ^1^H NMR (CDCl_3_) *δ* 9.59 (s, 1H), 8.53 (d, *J* = 5.9 Hz, 1H), 7.68 (dd, *J* = 7.9 Hz, *J* = 0.8 Hz, 1H), 7.51 (dd, *J* = 7.9 Hz, *J* = 0.8 Hz, 1H), 7.00 (d, *J* = 7.9 Hz, 1H), 3.07 (s, 4H), 2.58 (s, 3H), 1.82–1.88 (m, 4H), 1.65–1.78 (m, 2H).

To a stirred solution of **105** (0.169 g, 1.25 mmol) in MeOH (7.5 mL) at 0 °C was added NaCNBH_3_ (0.235 g, 3.73 mmol), and the mixture stirred at this temperature for 10 min·BF_3_.OEt_2_ (0.46 mL, 3.73 mmol) was then added and the mixture stirred at 0 °C for 1 h, then at reflux for 3 h. The reaction was cooled and quenched with sat. NaHCO_3_, and the solvent removed under reduced pressure. The residue was partitioned between EtOAc and water, and the organic phase was dried, filtered and evaporated. Column chromatography (19:1, DCM:MeOH) afforded 5-methyl-8-(piperidin-1-yl)-1,2,3,4-tetrahydroisoquinoline (**106**) as a brown oil (0.171 g, 100%), which was used directly. ^1^H NMR (CDCl_3_) *δ* 7.01 (t, *J* = 8.0 Hz, 1H), 6.85 (d, *J* = 8.0 Hz, 1H), 4.09 (s, 2H), 3.24 (t, *J* = 6.2 Hz, 3H), 2.17 (s, 3H), 1.65–1.78 (m, 2H), 1.49 (s, 2H), 2.67–2.72 (m, 6H), 2.17 (1H).

To a solution of **106** (0.094 g, 0.408 mmol) and 2-(bromomethyl)-5-(4-chloro-2-methylphenyl)pyridine (**84**) (0.133 g, 0.449 mmol) in DMF (8 mL) was added K_2_CO_3_ (0.068 g, 0.492 mmol) and the resultant mixture stirred at room temperature for 5 h. The reaction was then diluted with EtOAc (50 mL) and washed with water (5 × 10 mL), and the organic layer was dried and evaporated. Column chromatography (2:1, x4:EtOAc) afforded compound **55** as a tan solid (0.059 g, 32%); mp 96–98 °C. ^1^H NMR (CDCl_3_) *δ* 8.52 (d, *J* = 1.5 Hz, 1H), 7.57–7.62 (m, 2H), 7.23–7.32 (m, 2H), 7.15 (d, *J* = 7.8 Hz, 1H), 6.99 (d, *J* = 8.0 Hz, 1H), 6.82 (d, *J* = 8.0 Hz, 1H), 3.92 (s, 2H), 3.78 (s, 2H), 2.86 (t, *J* = 5.4 Hz, 2H), 2.71–2.81 (m, 6H), 2.25 (s, 3H), 2.18 (s, 3H), 1.55–1.62 (m, 4H), 1.49 (s, 2H); Anal. (C_28_H_32_ClN_3_) C, H, N.

## Declaration of Competing Interest

The authors declare that they have no known competing financial interests or personal relationships that could have appeared to influence the work reported in this paper.
